# Daily Reportable Disease Spatiotemporal Cluster Detection, New York City, New York, USA, 2014–2015

**DOI:** 10.3201/eid2210.160097

**Published:** 2016-10

**Authors:** Sharon K. Greene, Eric R. Peterson, Deborah Kapell, Annie D. Fine, Martin Kulldorff

**Affiliations:** New York City Department of Health and Mental Hygiene, Queens, New York, USA (S.K. Greene, E.R. Peterson, D. Kapell, A.D. Fine);; Brigham and Women’s Hospital, Harvard Medical School, Boston, Massachusetts, USA (M. Kulldorff)

**Keywords:** communicable diseases, disease clustering, outbreaks, detection, epidemiology, surveillance, foodborne diseases, Legionella, legionellosis, Shigella, shigellosis, zoonoses, bacteria, New York City, New York, USA

## Abstract

Each day, the New York City Department of Health and Mental Hygiene uses the free SaTScan software to apply prospective space–time permutation scan statistics to strengthen early outbreak detection for 35 reportable diseases. This method prompted early detection of outbreaks of community-acquired legionellosis and shigellosis.

The Bureau of Communicable Disease (BCD) at the New York City Department of Health and Mental Hygiene (DOHMH) monitors and investigates >70 reportable diseases among the city’s 8.49 million residents. Each day, healthcare providers and laboratories submit ≈1,000 communicable disease reports to BCD. Clusters (significant increases in observed vs. expected cases) and outbreaks (clusters believed to be associated with a common infection source) are detected through several methods, including notification by astute healthcare providers and by applying the modified historical limits method to detect increases in disease counts during the previous 4 weeks (*1*). This temporal analysis is applied weekly citywide and for each of 5 boroughs and 42 neighborhoods.

Cluster detection methods have been applied to syndromic data sources (e.g., emergency department visits) since the early 2000s (*2*,*3*). Less extensively described is cluster detection using reportable disease data, which reflect specific laboratory-confirmed diagnoses, contain patient home addresses, and may include illness onset dates and work addresses collected during patient interviews and medical record reviews. Other public health practitioners have applied purely temporal prospective cluster detection methods to reportable disease data (*4*,*5*) or conducted proof-of-concept spatiotemporal prospective analyses (*6*,*7*). However, published descriptions of actual prospective application of spatiotemporal methods to reportable diseases are rare (*8**,**9*), suggesting lack of widespread adoption among public health officials. We describe BCD’s experience with automated daily reportable disease spatiotemporal cluster detection using prospective space–time permutation scan statistics (*3*) in SaTScan (*10*) during February 2014–September 2015, highlighting instances in which findings guided public health action.

## The Study

For 35 reportable communicable diseases for which cluster detection could inform programmatic activities (*1*), we analyzed disease counts for patients of all ages combined. For amebiasis, cryptosporidiosis, and giardiasis, for which outbreaks among young children are of particular interest, additional analyses were restricted to disease counts among patients <5 years of age, for 38 total daily analyses.

In BCD’s application, the space–time permutation scan statistic detects disease clusters in space–time cylinders centered on every census tract centroid; the circular base represents space (maximum geographic cluster size of 50% of all reported cases), and the height represents time (maximum temporal window length of 30 days, for most diseases). For each cylinder, a likelihood ratio–based test statistic is calculated. The test statistic is considered elevated if the observed disease count during the time window in census tracts with centroids inside the cylinder’s circular base exceeds the expected number of cases, which is a function of 1) the case count in the circle during a baseline period (which accounts for any purely geographic variations in disease occurrence, diagnosis, and reporting) and 2) the total case count citywide during the time window (which accounts for citywide purely temporal patterns, such as seasonality or secular trends) (*3*). The cylinder with the maximum test statistic is the cluster least likely to be due to chance under the null hypothesis that the same process generated disease counts inside and outside the cylinder.

To create a simulated dataset, cases’ dates are randomly shuffled and assigned to the original census tracts. The maximum statistic for each simulated dataset is calculated in the same way as for the observed dataset. For each disease, this process is repeated daily 999 times. The maximum value for the observed dataset is ranked among the 999 trial maxima. A p value (range 0.001–1) is derived from this ranking; p = 0.001 represents the highest significance relative to the permutation trials. The Monte Carlo approach to deriving significance by using repeated trials, each permuting observed data attributes, is designed to control for multiple testing.

A recurrence interval (RI) is calculated as the reciprocal of the p value and represents the number of days of daily surveillance required for the expected number of clusters at least as unusual as the observed cluster to be equal to 1 by chance (*11*). We defined a signal as any cluster with an RI >100 days; that is, during any 100-day daily analysis period, the expected number of clusters at least as unlikely as the current cluster is 1.

We developed a SAS program (SAS Institute, Inc., Cary, NC, USA) to generate case and parameter files (Table 1), read in a coordinate file of census tract centroids, invoke SaTScan in batch mode, read analysis results back into SAS for further processing, and output files to secured folders. For any signals, the program also generated emails notifying BCD leadership and staff responsible for follow-up (Technical Appendix).

**Table 1:** Case file specifications for routine reportable disease analyses in New York City, New York, using the prospective space–time permutation scan statistic

Feature	Selection	Notes
Geographic aggregation	Census tract (defined using US Census 2000 boundaries) of residential address at time of report*	The less data are spatially aggregated, the more precisely areas with elevated rates can be identified. New York City has 2,216 census tracts in an area of 305 square miles.
Date of interest for analysis	Event date, defined using hierarchy of onset date → diagnosis date (collection date of first specimen testing positive) → report date → date event created in surveillance database	Defining reportable disease clusters according to when case-patients became ill is preferred. However, onset date is missing for most case-patients who have not yet been interviewed, and each case needs a date to be included in analysis. Thus, the best available proxy for onset date is used. Because we use daily data (rather than weekly, monthly, or yearly data), the time precision is specified as day on the SaTScan (http://www.satscan.org/) input tab. The time precision parameter indicates the temporal resolution of the data in the case file.
Study period	1 y for most diseases, ending the day before analysis†	One year is a reasonable choice, balancing the need for a period long enough to establish a stable local baseline for each spatial unit, yet short enough to avoid variable secular trends (e.g., geographically different increases in the underlying population of a spatial unit). Analyses are run each morning using data with event dates through the previous day.
Case inclusion criteria	Include all reported cases, regardless of current status (e.g., confirmed, probable, suspected, pending, noncase)†	Depending on the disease, cases initially might be assigned a transient pending status and, upon investigation, be reclassified as a case (confirmed, probable, or suspected) or a noncase. Timeliness is preserved by analyzing all reported cases, including noncases and pending cases, regardless of whether they ultimately will be confirmed. By analyzing all reported cases, case inclusion criteria are consistent across the study period. If instead the case file were restricted to confirmed and pending cases, then analyses would be biased toward false signaling, as some cases with an initial pending status will be ultimately reclassified after investigation as a noncase. This reclassification process is complete for the baseline but ongoing for the current period of interest (*1*), and the speed of reclassification might vary geographically.
Day-of-week variable	Include a variable that indicates the day of the week (1–7)	The analysis automatically adjusts for day-of-week effects but not for space by day-of-week interaction. Including this variable in the SaTScan case file accounts for how the daily pattern of health-seeking behavior and diagnoses might vary geographically.

*Exception to residential address at time of report: if the residential address is not geocodable (e.g., because the case-patient is not a resident of the city or because a post office box is reported instead of a street address), then the geocoded work address, if available, is substituted. †For exceptions, see online Technical Appendix (http://wwwnc.cdc.gov/EID/article/22/10/16-0097-Techapp1.pdf).

This automated analysis detected the second largest US outbreak of community-acquired legionellosis (*12*), identifying a cluster of 8 cases centered in the South Bronx on Friday, July 17, 2015 (RI = 500 days) (Figure), before any human public health monitor noticed it. On Monday, July 20, an increase in cases was independently noticed by BCD staff members routinely investigating individual cases, and on July 21, an infection-control nurse working in the outbreak area called BCD to report an increase. The DOHMH and state and federal partners conducted an extensive epidemiologic, environmental, and laboratory investigation to identify and remediate the outbreak source, a cooling tower.

**Figure:**
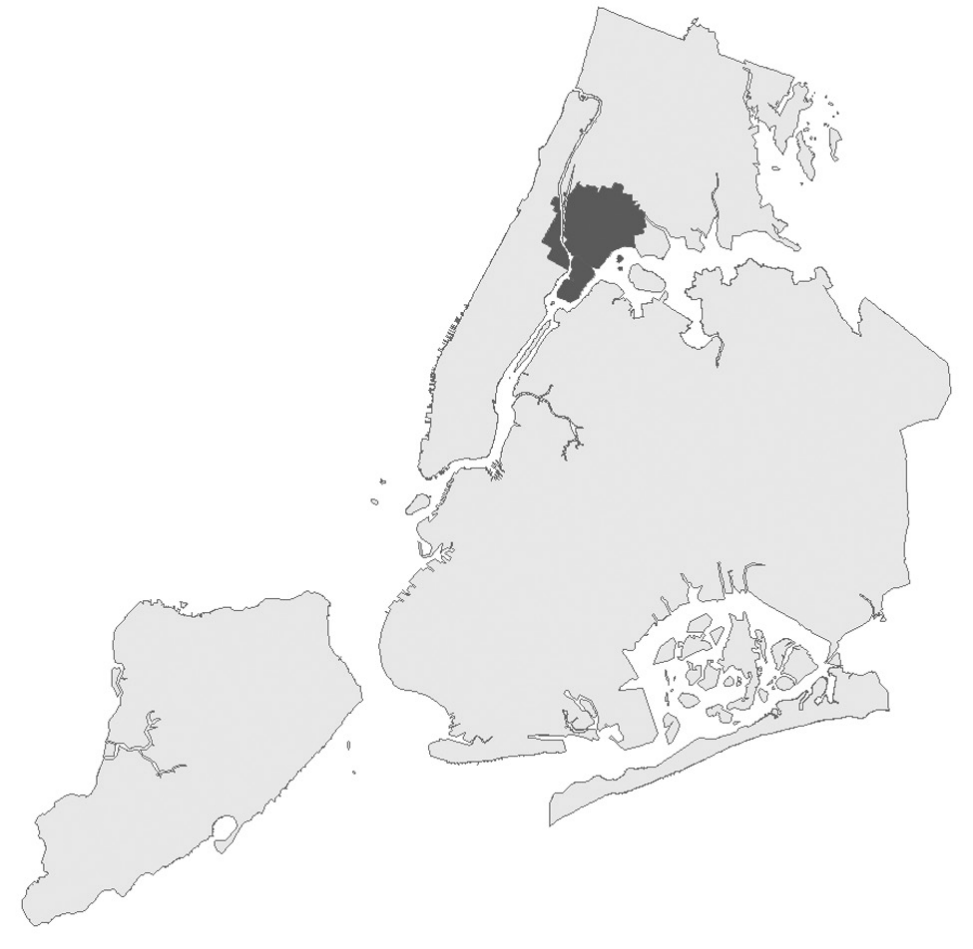
Automated output from spatiotemporal analysis on July 17, 2015, indicating a cluster (dark gray) of 8 legionellosis cases over 8 days centered in the South Bronx, New York City, New York, USA. In subsequent days, this cluster expanded in space and time into the second largest US outbreak of community-acquired legionellosis.

A shigellosis outbreak among the observant Jewish community in Brooklyn (*13*) began in late October 2014 and was detected with 9 cases on November 14, 2014 (RI = 333 days). BCD does not routinely investigate individual shigellosis reports, so automated analysis alone prompted early outbreak identification. Shigellosis outbreaks within this community occur cyclically and have been linked to daycare and preschool attendance (*14*). Starting in mid-November, BCD staff visited community schools, daycare centers, and health fairs to promote appropriate handwashing. The outbreak subsided by mid-March 2015. Other clusters prompting investigations included legionellosis (Queens, April–May 2015) and campylobacteriosis (Brooklyn, October 2014). During a 1-year period, 28 unique signals were observed across 15 diseases (Table 2), which staff perceived as a reasonable number for investigation.

**Table 2:** Signaling rates at 3 recurrence interval thresholds for 35 reportable diseases under surveillance in New York City, New York, USA, 2014–2015*

Disease	Annual no. cases‡	No. signals during 365 d of prospective surveillance†		
		Recurrence interval >365 d§	Recurrence interval >100 d	Recurrence interval >30 d
Amebiasis	476	0	1.2	4.3
Babesiosis	57	0	0	0
Campylobacteriosis	1,663	0.6	0.6	4.9
Chikungunya	171	0.6	1.8	3.1
Cholera	0	0	0	0
Cryptosporidiosis	135	0	0	0.6
Cyclosporiasis	51	0	0	1.2
Dengue	57	0	0	1.8
Encephalitis	2	0	0	0
Giardiasis	871	1.2	1.8	5.5
Hemolytic uremic syndrome	4	0	0	0
Hepatitis A	78	1.9	1.9	5.8
Acute hepatitis B	51	0.6	1.2	3.7
Hepatitis D	0	0	0	0
Hepatitis E	0	0	0.6	0.6
Human granulocytic anaplasmosis	51	0.6	0.6	0.6
Human monocytic ehrlichiosis	8	0	0.6	0.6
Invasive group A *Streptococcus* disease	263	0	0	1.8
Invasive group B *Streptococcus* disease	33	0.6	1.2	2.4
Invasive *Haemophilus influenzae* disease	97	0	0	1.8
Invasive *Streptococcus pneumoniae* disease	647	0	1.2	1.8
Legionellosis	434	9.1	9.1	11.4
Listeriosis	34	0	0	0.6
Malaria	187	0.6	1.8	4.3
Meningococcal disease	8	0	0	0.6
Noncholera *Vibrio* spp. infection	18	0	0	0
Paratyphoid fever	11	0	0	0
Rickettisalpox	9	0	0	0
Rocky Mountain spotted fever	6	0	0	2.4
Shiga toxin–producing *Escherichia coli*	96	0	0	0
Shigellosis	806	1.8	1.8	6.1
Typhoid fever	31	0	1.9	3.8
Vancomycin-intermediate *Staphylococcus aureus* infection	28	0	0	0
West Nile virus disease	19	0	0	0
Yersiniosis	25	0	0	0
Total signals across all diseases under surveillance	NA	17.8	27.6	69.8

*Signals were detected by using the prospective space–time permutation scan statistic. NA, not applicable. †A signal for a particular disease was defined as unique if the first most likely cluster on a particular day did not encompass any of the same census tracts as the first most likely cluster on the prior day. The signaling rate for most diseases was based on 598 d of surveillance (February 10, 2014–September 30, 2015). For 5 diseases, the signaling rate was based on a shorter surveillance period to reflect analytic adjustments: hepatitis A, paratyphoid fever, and typhoid fever (190 d under surveillance after extending to a 60-d maximum temporal cluster size); legionellosis (160 d under surveillance after excluding unresolved cases); and Shiga toxin–producing *E. coli* (21 d under surveillance after excluding cases with only a positive multiplex PCR gastrointestinal panel test). ‡Confirmed, probable, and suspected cases among residents with event dates October 1, 2014–September 30, 2015. §The signal was detected at the lower ≥100-d threshold on the same day for 50% of the signals, 1 d earlier for 19% of signals, 2 d earlier for 19% of signals, 3 d earlier for 6% of signals, and 7 d earlier for 6% of signals.

Not all detected clusters were actionable. No public health response was conducted for an amebiasis cluster (Manhattan, April 2015; RI = 143 days) consisting of 6 men (34–49 years of age) diagnosed within a 12-day period and residing within a 0.35-mile radius because no case-patients were identified as food handlers or daycare workers. A public health response also was not conducted for a giardiasis cluster (Bronx, April 2015; RI = 1,000 days) that consisted of 6 household members who acquired the infection during international travel. Investigators were interested in being notified of and following such clusters over time, even if they ultimately were not actionable or verified as true outbreaks.

## Conclusions

Several outbreaks in New York City, New York, were detected by daily automated spatiotemporal analyses. Early cluster detection facilitated prioritization of individual case investigations, outbreak recognition and investigation, provider and community outreach, and timely intervention to limit sickness and death. This method has proven particularly useful for identifying and monitoring outbreaks of shigellosis (*6*,*8*,*9*) and legionellosis and might be useful for monitoring additional diseases with outbreak potential, including pertussis, syphilis, and tuberculosis.

Key to the system’s success is a strong informatics infrastructure, especially electronic laboratory reporting and near real-time geocoding of surveillance data. Other facilitators include a powerful statistical disease surveillance methodology, knowledgeable epidemiologists to interpret signals, and adequate outbreak investigation resources.

These methods could be useful to other health departments receiving more reports than can be rapidly reviewed manually. State health departments could consider conducting similar analyses to detect clusters spanning multiple jurisdictions.

Technical Appendix Supplemental information, SAS code, and sample output for daily reportable disease spatiotemporal cluster detection, New York City, New York, USA.
