# Supplementary material for: Daily Reportable Disease Spatiotemporal Cluster Detection, New York City, New York, USA, 2014–2015
Source: Emerg Infect Dis. 2016 Oct;22(10):1808–12. doi: 10.3201/eid2210.160097 (PMC5038417; doi:10.3201/eid2210.160097)
Supplement: Technical Appendix — Supplemental information, SAS code, and sample output for daily reportable disease spatiotemporal cluster detection, New York City, New York, USA. [file 16-0097-Techapp-s1.pdf]

Article DOI: http://dx.doi.org/10.3201/eid2210.160097

Daily Reportable Disease Spatiotemporal Cluster Detection, New York City, New York, USA, 2014–2015

Technical Appendix

This technical appendix includes

1. Sample output produced for signals above the designated recurrence interval (RI) threshold

2. Exceptions to case file specifications in main text Table 1

3. Explanation of analysis parameter settings (Technical Appendix Table 1)

4. Summaries of first ten days of signals detecting true outbreaks (Technical Appendix Tables 2 and 3)

5. Explanation of choice of space-time permutation probability model

6. Notes on system maintenance

7. Data structure

8. Annotated SAS code (SAS v.9.2, SAS Institute, Cary, NC, USA).

Sample Output: First signal detecting a shigellosis (SHG) outbreak—Williamsburg, Brooklyn, New York City, November 2014

The following is a sample of the output produced when a cluster identified by SaTScan exceeds the designated RI threshold. Output is generated automatically in secure archive folders organized by date of analysis and includes a map indicating the location of the cluster(s), cluster summary information, and a linelist of cases residing in the cluster. For ongoing clusters, a report is only generated when at least one additional confirmed, probable, suspected, or pending case had been added to the cluster since the last output. Output elements not shown here are (a) a

linelist of cases not in the cluster by primary address who have a secondary address that falls within the geographic bounds of the cluster, and (b) a linelist of cases in the cluster who have an additional address or addresses within the geographic bounds of the cluster. These linelists are only generated when supplemental events or addresses exist. Case coordinates in the map output have been randomized within the bounds of the cluster to protect confidentiality and do not reflect the true addresses of cases.

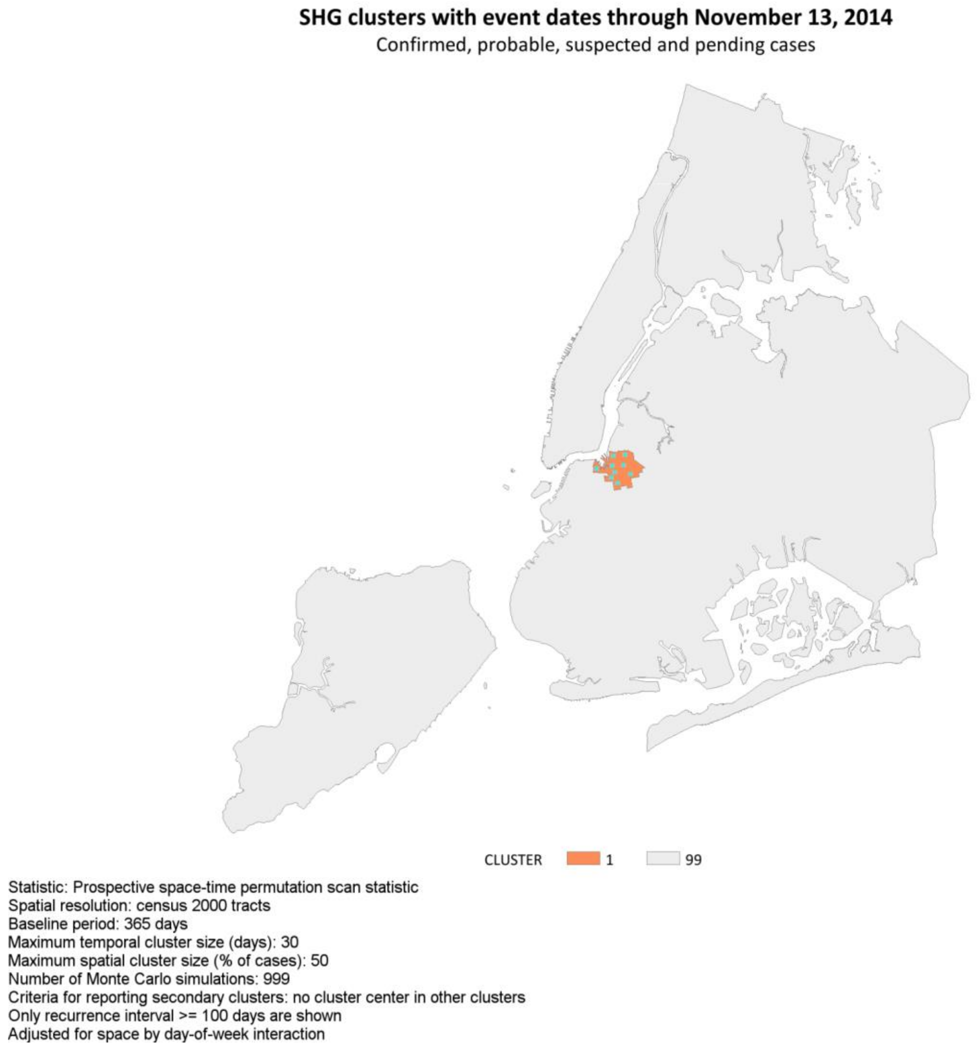

Sample automated table: Cluster summary information

Cluster	Start date	End date	No. days in cluster	Radius, miles	Observed/ expected	Recurrence interval, days*
1	2014 Oct 20	2014 Nov 13	25	0.68	6.53	333

Sample automated table: Linelist of cases residing in the cluster

Cluster	New event	Event ID	Disease status	Invest status	Event date, 2014	Age	Address	Borough	Zip code	United Hospital Fund neighborhood	Census tract
1	Yes	XXXXXXX	Conf	NN	Oct 20	X	XXXXXXXXX	BK	XXXXX	X	XXXXXXXXXXX
	Yes	XXXXXXX	Conf	NN	Oct 21	XX	XXXXXXXXX	BK	XXXXX	Y	XXXXXXXXXXX
	Yes	XXXXXXX	Conf	NN	Oct 21	X	XXXXXXXXX	BK	XXXXX	X	XXXXXXXXXXX
	Yes	XXXXXXX	Conf	NN	Oct 24	X	XXXXXXXXX	BK	XXXXX	Z	XXXXXXXXXXX
	Yes	XXXXXXX	Conf	NN	Oct 27	X	XXXXXXXXX	BK	XXXXX	Z	XXXXXXXXXXX
	Yes	XXXXXXX	Conf	NN	Nov 03	X	XXXXXXXXX	BK	XXXXX	Z	XXXXXXXXXXX
	Yes	XXXXXXX	Conf	NN	Nov 05	XX	XXXXXXXXX	BK	XXXXX	Y	XXXXXXXXXXX
	Yes	XXXXXXX	Conf	NN	Oct 30	.	XXXXXXXXX	BK	XXXXX	Z	XXXXXXXXXXX
	Yes	XXXXXXX	Conf	NN	Nov 09	X	XXXXXXXXX	BK	XXXXX	X	XXXXXXXXXXX

BK, Brooklyn Conf, confirmed; Invest, investigation; NN, not needed.

Exceptions to Case File Specifications in Main Text Table 1

1. Exceptions to the 1-y study period: for analyses with a longer maximum temporal window, the study period is extended accordingly so the temporal window of interest does not constitute a disproportionately high fraction of the study period. For the 60-d maximum temporal window, the study period is extended to 1.5 y. For shigellosis, outbreaks occur cyclically and have prolonged person-to-person transmission. The study period was extended to 2 y so that as cases in ongoing outbreaks shift as time passes from the temporal window of interest to the baseline, they do not constitute a disproportionately high fraction of the study period. Including true outbreak-associated cases in the baseline period of the case file can bias prospective analyses, making it more difficult to detect an elevated number of diagnoses in an area with a past outbreak. To minimize this bias, for example, after a large legionellosis outbreak in the South Bronx (*1*), all cases citywide with event dates of July 8–August 3, 2015, were removed from the case file. Including nosocomial outbreak-associated cases in the temporal period of interest in the case file can make it more difficult to detect a simultaneous outbreak of community-associated cases. To minimize this bias, after an outbreak of legionellosis in a

nursing home, the 7 cases for patients residing in this home during the outbreak period were removed from the case file.

2. Exceptions to including all reported cases: Including all cases assumes that the overall reporting volume is constant over time. This assumption can be violated by geographic changes in laboratory testing practices. For legionellosis, 1 case status (unresolved) is reserved for events for which the only laboratory report is an antibody test; such events do not meet the case definition. Before excluding this case status, several false legionellosis signals occurred. One hospital laboratory adopted a new culture-independent diagnostic test for a panel of pathogens causing gastrointestinal illness. Before excluding cases where the only laboratory report was from this panel, several clusters of Shiga toxin–producing *Escherichia coli* were attributable to reports from this facility using this diagnostic test, but the cases did not meet the case definition.

Technical Appendix Table 1. Analysis parameter settings for routine reportable disease analyses in New York City using the prospective space-time permutation scan statistic.

Parameter	Parameter setting	Notes
Analysis type	Prospective space-time	For timely cluster detection, prospective (rather than retrospective) analyses are used, evaluating only the subset of possible clusters that encompass the last day of the study period. Such clusters do not necessarily require the existence of cases at the very end of the study period.<br>To detect acute, ongoing, localized disease clusters, space-time analyses (rather than purely temporal or purely spatial analyses), are used.
Model type	Space-time permutation	The space-time permutation probability model has several advantages over other models developed for count data, including requiring only case data and not requiring the assumption that the probability of being diagnosed and reported as a case is independent of location of patient residence.
Scan areas	High rates	For disease cluster detection, areas with high (rather than low) rates are of interest. Separate quality control measures are in place to detect unusual drop-offs in disease reporting.
Time aggregation and length	1 d	This setting must equal the interval between prospective analyses. For daily analyses, data must be aggregated into units of one day, corresponding with the daily resolution of data in the case file.
Maximum spatial cluster size	50% of all cases during the study period	The option that imposes the fewest assumptions is to allow the cluster to expand in size to include up to 50% of all cases during the study period. Forcing clusters to be smaller than 50% (or restricting in terms of geographic size by setting a maximum circle radius) can be arbitrary and lead to selection bias.
Maximum temporal cluster size	30 d*	Thirty days is long enough to encompass most cases in point-source clusters, which typically span a few days or weeks, depending on the pathogen. Thirty days may not be long enough for diseases with long incubation periods, extended propagated transmission, or intermittent common-source outbreaks.
Maximum number of Monte Carlo replications	999	The number of replications must be at least 999 for adequate power. To minimize computing time across the many diseases being independently analyzed each day, we chose not to increase to 9,999 replications.
Secondary cluster reporting criteria (output parameter)	No cluster centers in other clusters	Any disease may have multiple active clusters at any moment, so secondary clusters should be reviewed. By reviewing clusters with no cluster centers in other clusters (rather than no, or more geographic overlap), secondary clusters with some overlap can be detected.

*Exceptions to the 30-d maximum temporal cluster size: The maximum temporal cluster size is extended to 60 d for Shiga toxin-producing *E. coli*, hepatitis A, typhoid fever, and paratyphoid fever. FoodCORE performance metrics define a cluster as two or more cases with an indistinguishable PFGE pattern in 60 d (U.S. Centers for Disease Control and Prevention. Foodborne Diseases Centers for Outbreak Response Enhancement: Salmonella, Shiga toxin-producing *Escherichia coli*, and Listeria (SSL) Metrics. 2015 [cited 2015 Sep 24]. http://www.cdc.gov/foodcore/ssl-metrics.html).

Technical Appendix Table 2. First ten days of signals detecting a legionellosis outbreak — South Bronx, New York City, July 2015*

Signal date, 2015	Cluster start date, 2015	Days in cluster	Radius, miles	Observed, all case statuses†	Expected, all case statuses	Observed/ expected	Recurrence interval, d
Friday, Jul 17	Jul 9	8	1.57	8	0.83	9.66	500
Saturday, Jul 18	Jul 12	6	1.05	6	0.31	19.26	1,000
Sunday, Jul 19	Jul 12	7	1.05	6	0.31	19.12	1,000
Monday, Jul 20	Jul 12	8	1.05	6	0.31	19.12	1,000
Tuesday, Jul 21	Jul 8	13	2.12	12	2.22	5.42	500
Wednesday, Jul 22	Jul 9	13	2.38	13	2.17	5.99	1,000
Thursday, Jul 23	Jul 8	15	2.40	17	2.84	5.98	1,000
Friday, Jul 24	Jul 8	16	2.47	21	4.45	4.72	1,000
Saturday, Jul 25	Jul 8	17	2.01	22	5.47	4.02	1,000
Sunday, Jul 26	Jul 8	18	2.47	21	4.72	4.45	1,000

*NYC DOHMH. Health Alert Network. Alert #21: Increase in Legionnaires' disease in the Bronx. New York City Department of Health and Mental Hygiene: New York. 2015 [cited 2015 Sep 24]. https://a816-health30ssl.nyc.gov/sites/nychan/Lists/AlertUpdateAdvisoryDocuments/HAN_LegionellaSouthBronx.pdf.
†The number of observed cases in the most likely cluster can increase or decrease on consecutive days, depending on how the cluster's spatial and temporal extent change as cases are newly diagnosed, reported, and interviewed to determine an onset date.

Technical Appendix Table 3. First ten days of signals detecting a shigellosis outbreak—Borough Park and Williamsburg, Brooklyn, New York City, November 2014*

Signal date, 2014	Cluster start date, 2014	No. days in cluster	Radius, miles	No. observed	No. expected	Observed/ expected	Recurrence interval, d
Friday, Nov 14	Oct 20	25	0.68	9	1.38	6.53	333
Saturday, Nov 15	Oct 20	26	0.68	9	1.38	6.53	250
Sunday, Nov 16	Oct 20	27	1.06	13	2.61	4.98	500
Monday, Nov 17	Oct 18	30	1.53	14	3.49	4.01	111
Tuesday, Nov 18	Oct 20	29	1.06	15	3.19	4.70	1,000
Wednesday, Nov 19	Oct 20	30	1.06	16	3.43	4.66	1,000
Thursday, Nov 20	Oct 21	30	0.86	13	2.67	4.88	500
Friday, Nov 21	Oct 24	28	0.97	12	2.45	4.89	500
Saturday, Nov 22	Oct 24	29	0.97	13	2.64	4.92	1,000
Sunday, Nov 23	Oct 24	30	0.86	14	3.08	4.55	1,000

*NYC DOHMH. Health Alert Network. Alert #39: Outbreak of shigellosis in Borough Park and Williamsburg. New York City Department of Health and Mental Hygiene: New York. 2014 [cited 2015 Sep 24]. https://a816-health30ssl.nyc.gov/sites/nychan/Lists/AlertUpdateAdvisoryDocuments/HAN_Shigella.pdf

Rationale for Space-Time Permutation Instead of Poisson or Bernoulli Probability Models

For count data such as reportable disease events, three probability models are available for spatiotemporal analyses using SaTScan (*2*,*3*). The Poisson model requires geographically aggregated cases and population counts, and the null hypothesis assumes that the probability of being observed as a case is not spatially dependent. However, if case-patients residing in one part of a jurisdiction are more likely to present for care (e.g., due to insurance coverage or access to care) and be tested, diagnosed, and reported with a disease, then the null hypothesis can be violated. The Bernoulli model requires cases and controls with temporal and spatial attributes; e.g., controls might be defined as cases of a different disease. The null hypothesis assumes that the ratio of cases and controls is constant across space and time, but if the cases and controls have different seasonal patterns or day-of-week effects, then the null hypothesis can be violated.

Passively reported disease surveillance data likely violate the assumptions required for the Poisson and Bernoulli models. Thus, we selected the space-time permutation probability model, which requires only case data and automatically adjusts for any purely geographic and any citywide purely temporal trends.

System Maintenance

The SAS program was run automatically by Microsoft Task Scheduler every morning, using data with event dates through the end of the prior day. The entire program analyzing all diseases takes ≈40–60 minutes to run each morning on a dedicated computer (Quad Core i7 CPU with 16GB RAM); the exact run time varied depending on disease volume and whether there was a signal. Analyses were completed by open of business to inform activities for that workday.

For each disease regardless of signal status, the daily case input file and SaTScan results files were archived. Cluster summary information (one row per disease per day) was appended to a cumulative SAS dataset to track how clusters strengthened or weakened over time.

Timely cluster detection is dependent on up-to-date data. Delays resulted when diseases were reported by telephone or fax instead of electronically and reports were still awaiting data entry at the time of analysis. Relevant spatial and temporal data elements obtained from case investigation (e.g., work address and onset date) also needed to be entered quickly.

Strong data quality control procedures must be maintained to avoid missed and false signals. Patient addresses that were not geocodable (e.g., due to typos) were reviewed and corrected if possible to keep those cases in analysis and retain power. Multiple disease reports for the same patient were de-duplicated to avoid false signals.

Information technology problems need to be anticipated to avoid disrupting analyses. On a few occasions, security updates and forced computer reboots interfered with programs scheduled to run automatically. Live, completely refreshed data were not always available. In addition, electronic reporting must be continuously maintained for all clinical laboratories, including during transitions when files are upgraded to meet Meaningful Use requirements. The assumption of no spatial variation in data accrual delays can be violated if a laboratory serving patients in one geographic area encounters difficulties in electronically transmitting reports.

Interpreting signals can be challenging. With 35 diseases under surveillance and a signaling threshold of RI ≥100 days, we could expect to see over 100 false signals a year due to chance. Our observed signaling rate was much lower, because some diseases had very low disease counts. The number of signals could easily be reduced by setting a higher threshold, but that could lead to missed or delayed signals of public health importance. Lower or higher signaling thresholds could have been used, considering the seriousness of an outbreak of a particular disease and weighing the relative costs of investigating false signals versus not responding to true signals. A higher signaling threshold of RI ≥365 days would have yielded 18 unique (36% fewer) signals across 10 diseases, and a lower signaling threshold of RI ≥30 days would have yielded 70 unique (153% more) signals across 23 diseases (main text Table 2).

Rather than using a fixed statistical threshold to launch a formal outbreak investigation, it is better to rely on experienced epidemiologists to weigh several factors, including the type of disease, the patient population, and the RI magnitude. Signals suggest excess disease activity and are intended to facilitate investigations, but provide only approximate spatial and temporal boundaries for a suspected outbreak. Some patients within the cluster likely represent background, non-outbreak-associated cases, while some patients outside the cluster may have had the outbreak-causing exposure. In addition, a geographically large cluster may have a less clear public health action compared with highly spatially focused clusters.

Analytic adjustments were warranted in response to false, delayed, and difficult-to-interpret signals. False signals were attributable to the inclusion of legionellosis cases with only antibody test results and Shiga toxin-producing *E. coli* cases with only culture-independent diagnostic test results. These reports did not meet the case definitions and reflected the adoption of specific tests by specific facilities rather than true disease increases. Case files were modified to exclude such cases (main text Table 1). A legionellosis outbreak in the East Bronx in September 2015 was detected later than it might have been, because the only spatial element included in analysis initially was the home address of NYC residents, whereas the outbreak also affected those who worked or attended programs but did not live in the area. The case file was modified to include the NYC work address obtained from case investigation if a NYC home address was unavailable (main text Table 1). In addition, in response to heightened public concern following a large community-acquired legionellosis outbreak, the RI signaling threshold

was lowered from 100 to 30 days to facilitate earlier cluster detection at the possible expense of increased false signaling.

Several signals were difficult to interpret due to the influence of past outbreaks in the study period. Following a nosocomial legionellosis outbreak in a nursing home, the individual outbreak-associated cases were excluded from the case file. Following large community-associated legionellosis outbreaks, all cases citywide during each outbreak period were excluded from the case file. In addition, a shigellosis outbreak was so prolonged that after 3 months, the center of the cluster appeared to migrate to a different part of the city, despite continued excess shigellosis activity in the original outbreak location. The case file was modified to double the study period so that as older cases in the ongoing outbreak shifted from the temporal cluster window of interest into the baseline period, they would not unduly influence the baseline (main text Table 1).

Data Structure

A case file was generated daily from BCD’s surveillance database (Maven, Consilience Software, Austin, TX) for each disease according to the specifications in main text Table 1. For each reported disease case, the “event date,” (the illness onset date if available; otherwise the diagnosis date) was noted. Cases were geocoded to census tracts in near real-time by integrating Maven with a custom geocoding web service referencing the NYC Department of City Planning’s Geosupport Desktop Edition software (*4*). Each case text file included: census tract, number of cases, event date, and a day-of-week indicator.

A geographic coordinate file is also required for analysis in SaTScan. Cases were assigned to the centroid of their census tract, defined using Census 2000 boundaries and a Cartesian coordinate system. The coordinate file included: census tract, X-coordinate, and Y-coordinate.

Sample Structure for Input Dataset of Analysis Parameters, Named “diseaselist”:

Recurrence: Recurrence interval threshold (days)

MaxTemp: Maximum temporal cluster size (days)

MaxGeog: Maximum spatial cluster size (percent of cases in study period)

Baseline: Length of study period (days)

Montecarlo: Number of Monte Carlo replications

Lagtime: Number of days between day of analysis and end of study period

Disease_code	Recurrence	MaxTemp	MaxGeog	Baseline	Montecarlo	Lagtime	AgeGroup
AMB	100	30	50	365	999	1	5orYounger
AMB	100	30	50	365	999	1	AllAges
ARB	100	30	50	365	999	1	AllAges
…	…	…	…	…	…	…	…
YER	100	30	50	365	999	1	AllAges

Sample SAS Code for Running SaTScan Analyses and Producing Output

This code generates case and parameter files formatted for SaTScan for each disease, reads in the NYC census tract coordinate file, calls SaTScan in batch mode, reads in analysis results, creates output files when a cluster is identified above the RI threshold, and sends e-mails to Bureau of Communicable Disease (BCD) leadership and designated staff for follow-up. The code is partitioned into three files, which are presented here in the order they are run by SAS (5,6). The first file reads in a series of macros that establish SaTScan input, analysis, and output parameters; call SaTScan in batch mode; flag new clusters above the RI threshold; and generate figures and linelists for output. The second file creates an event-level dataset of all diseases and runs iteratively over a list of designated disease-analysis parameter combinations to create disease-specific event-level datasets for analysis in SaTScan, then calls the macros to perform analyses and produce output files. The third file sends e-mails to alert staff to new and ongoing signal summary output and inform BCD data unit staff that the program has run successfully.

Jurisdictions seeking to adopt SaTScan for cluster detection should keep in mind that the parameters macro program presented here (*TractParam*) is only compatible with SaTScan version 9.1.1. If using a different version of SaTScan, it will be necessary to replace this code with parameters derived from the version of SaTScan to be used for analysis. This is done by setting input, analysis, and output parameters in the SaTScan user interface, saving the SaTScan session as a .txt file, and replacing the code in the body of the *TractParam* macro progam with the text of the saved .txt file. All lines of the substituted text will need to be enclosed in double

quotation marks and preceded by a forward slash, except the first line of each parameter group (e.g., [Input], [Analysis], etc.) which should be preceded by two forward slashes. The [System] parameter group can be excluded from the *TractParam* macro program. Be sure to make note of the locations where macro variables are used in the original text of the *TractParam* macro program, as these will need to be reinserted after substituting new parameters from the .txt file to retain the capacity to automate analyses over multiple disease-analysis parameter combinations. Potential adopters should also recognize the need to modify or remove sections of the code presented here that may not be relevant or applicable to their jurisdictions, including code removing STEC cases identified by BioFire testing, removing outbreak-associated disease events, replacing non-geocodable addresses with secondary addresses, restricting inclusion of encephalitis cases, identifying cases that do not match to a location in the geographic coordinates file, producing supplemental linelists in cluster output, and referencing NYC-specific (census tract, UHF neighborhood, borough) data elements.

When setting up SaTScan for automated cluster detection, it is advisable to separate into three steps. The first is preparing case files, reading them into SaTScan in batch mode for analysis, and importing SaTScan output into SAS. Preparing case files from disease surveillance data are the primary purpose of the analysis program (File 2) presented here, which requires referencing the analysis parameters dataset. Running SaTScan in batch mode is the focus of the first section of the macro programs file (File 1), and requires referencing the geographic coordinates file and calling the *TractParam* and *CallSatscan* macro programs. The user should be aware of the importance of setting the TXTFILES (temporary destination for case files), OUTPUT (temporary destination for SaTScan output files), and PARAM filepaths properly before attempting to run SaTScan in batch mode. If any of these folders do not exist in the SaTScan folder, they should be created, and the geographic coordinates file should be stored in the PARAM folder. The *callSatscan* macro program calls SaTScan in batch mode and imports SaTScan result files (col.dbf and gis.dbf) into SAS.

The second step is to format and evaluate SaTScan analysis results, and generate output for identified clusters. The relevant macro programs for this task are *Switch*, *Choropleth_Setup*, *Linelist_Setup*, *NewIndividuals*, *AddToList*, *MakeChoropleth*, *MakeClusterLinelist*, and *PersonLinelist*. To distinguish between new and previously identified clusters and disease

events, we maintain archive datasets (clusterhistory and satscanlinelist) of all identified clusters and events, which are referenced in several of the listed macro programs. A shapefile (.shp) of the area and geographic unit covered by the analysis is required to output a map of identified clusters. Our analysis is done on the census tract-level, but the code could be modified to accommodate analyses using different geographic units. Maps and linelists are output as .rtf files using the SAS Output Delivery System (ODS), which can be opened in Microsoft Word. Desired location for output should be specified in ODS RTF BODY and ODS RTF FILE statements in the *MakeChoropleth* macro program.

The third step is to distribute e-mail alerts to appropriate staff after the analysis has run and output is ready for review. To run successfully, the EMAILHOST and EMAILSYS options in SAS must be properly configured, and a dataset with disease codes and corresponding lists of emails for distribution (enclosed in quotation marks, space-delimited) must be available for reference. This e-mail distribution component of the code is optional and will not affect the results of the cluster detection analysis.

References

1. New York City Department of Health and Mental Hygiene. Health Alert Network. 2015 Alert 21: increase in Legionnaire’s disease in the Bronx. New York City Department of Health and Mental Hygiene: New York. 2015 [cited 2015 Sep 24]. https://a816-health30ssl.nyc.gov/sites/nychan/Lists/AlertUpdateAdvisoryDocuments/HAN_LegionellaSouthBronx.pdf

2. Kulldorff M; Information Management Services, Inc. SaTScan v9.1.1: software for the spatial and space-time scan statistics. 2015 [cited 2015 Sep 24]. http://www.satscan.org/

3. Kulldorff M. SaTScan user guide for version 9.4. 2015 [cited 2015 Sep 24]. http://www.satscan.org/techdoc.html

4. Geographic Systems Section, Information Technology Division, New York City Department of City Planning. Geosupport Desktop Edition software. Version 15C. New York, USA: The Department [cited 2015 Dec 4]. http://www1.nyc.gov/site/planning/data-maps/open-data/dwn-gde-home.page

5. Abrams AM, Kleinman KP. A SaTScan™ macro accessory for cartography (SMAC) package implemented with SAS® software. Int J Health Geogr. 2007;6:6. http://dx.doi.org/10.1186/1476-072X-6-6

6. Chen J-H, Weng C, Chang H-G. Using space-time scan statistic to detect pertussis and shigellosis outbreaks (poster). In: Abstracts of the 2013 Annual Conference of the Council of State and Territorial Epidemiologists; Pasadena, California; 2013 Jun 10-13; Abstract 1614 [cited 2015 Sep 18] http://www.cste2.org/annualconference/webpdfs/AC13AgendaAppWebPosterAwards.pdf

File 1: Macro Programs

```
/*************************************************************************/
/*    PROGRAM NAME: SaTScan_macros2                                      */
/*    DATE CREATED: February 10, 2014                                    */
/*    LAST UPDATED: November 4, 2015                                     */
/*    PROGRAMMERS: Deborah Kapell                                        */
/*                 Eric Peterson                                         */
/*                                                                       */
/*    PURPOSE: Reads in SaTScan macros                                   */
/*                                                                       */
/*************************************************************************/

ods results off;
/* Maven Business Intelligence (BI) Tables */
libname maven odbc database=MavenBCD_RPT owner=dbo;

/* SaTScan Folder */
libname satscan9 'C:\SaTScan9';

/* Analyst Tools Folder */
libname skt 'S:\BCD\COMDISshared\Analyst_of_the_week\Tools';

/* SAS Datasets of Maven Data for address histories */
libname mavensas 'S:\BCD\COMDISshared\Analyst_of_the_week\Maven\SAS_data';

/* SaTScan Output Archive Folder */
libname satscan 'S:\BCD\COMDISshared\Analyst_of_the_week\Maven\SaTScan';

/* Permanent SaTScan SAS Datasets: */
/*    Cluster history archive - all most likely clusters identified for */
/*          each disease on each day of analysis */
/*    SaTScan linelist archive - all events that have been identified as */
/*          part of a SaTScan cluster exceeding RI threshold. Used to    */
/*          determine if identified cluster has any new events */
/*    E-mail distribution list - BCD staff to be alerted to a new cluster */
/*          or event by disease */
libname support
'S:\BCD\COMDISshared\Analyst_of_the_week\Maven\SaTScan\SupportingFiles';


/* Set filepaths for output from batch runs of SaTScan. These locations    */
/*    are referenced in the TractParam and callSatscan macro programs,    */
/*    and in the Analysis Program file */
%LET TXTFILES =C:\SaTScan9\TXTFILES\;
%LET PARAM    =C:\SaTScan9\PARAM\;
```

```
%LET OUTPUT   =C:\SaTScan9\OUTPUT\;

/* SAS output window options */
ods noresults;
OPTIONS NOCENTER  nonumber  ls=140 ps=51 nodate;
options symbolgen;
options mlogic;
options mprint;
options source;
options source2;
options orientation=landscape;

/* Macro with input, analysis, and output parameters for SaTScan  */
%Macro TractParam;
"[Input Files]"
/"CaseFile=&&TXTFILES.casefile_&&disease_Code&i..&&maxTemp&i.._&&agegroup&i..
.txt"
/"ControlFile="
/"CoordinatesFile=&PARAM.TractNYCoord.txt"
/"GridFile="
/"UseGridFile=n"
/";                 Precision of case times (0=None, 1=Year, 2=Month, 3=day)"
/"PrecisionCaseTimes=3"
/";                                Coordinate type (0=Cartesian, 1=Lat/Long)"
/"CoordinatesType=0"


//"[Analysis]"
/";                      Analysis type (1=Purely Spatial, 2=Purely Temporal,"
/";                    3=Retrospective Space-Time, 4=Prospective Space-Time,"
/";                                     5=Spatial Variation/Temporal Trends,"
/";                   6=Prospective Purely Temporal,7=PurelySpatialMonotone)"
/"AnalysisType=4"
/";              Model type (0=Poisson, 1=Bernoulli, 2=Space-Time Permutation)"
/"ModelType=2"
/";                                   Scan areas (1=High, 2=Low, 3=High or Low)"
/"ScanAreas=1"
/";                                           Start date (YYYY/MM/DD)"
/"StartDate="startdt
/";                                           End date (YYYY/MM/DD)"
/"EndDate="EndDt
/";                                          Monte Carlo reps (0, 9, 999, n999)"
/"MonteCarloReps=&&montecarlo&i"


//"[Time Parameters]"
/";                          Interval units (0=None, 1=Year, 2=Month, 3=Day)"
/"IntervalUnits=3"
/";                              Interval length (positive integer) 6 weeks"
/"IntervalLength=1"
/";                          Prospective surveillance start date (YYYY/MM/DD)"
/"ProspectiveStartDate="ProspStartDate
/";               ; Time trend adjustment type (0=None, 1=Nonparametric,
  2=LogLinearPercentage, 3=CalculatedLogLinearPercentage,
4=TimeStratifiedRandomization)"
/"TimeTrendAdjustmentType=0"
```

```
/";                                         Time trend adjustment percentage 
(>-100)"
/"TimeTrendPercentage=0.000000"
/";                       Adjust for earlier analyses -- prospective only (y/n)"
/"AdjustForEarlierAnalyses=n"


//"[Scanning Window]"
/";                                         Max geographic size (<=50%)"
/"MaxGeographicSize=&&MAXGEOG&i"
/";                                 How max spatial size should be interpreted
      (0=Percentage, 1=Distance, 2=Percentage of max circle population file)"
/"MaxSpatialSizeInterpretation=0"
/";                                         Include purely temporal clusters 
(y/n)"
/"IncludePurelyTemporal=n"
/";                                         Max temporal size (<=90%)"
/"MaxTemporalSize=&&MAXTEMP&i"
/";       How max temporal size should be interpreted (0=Percentage, 1=Time)"
/"MaxTemporalSizeInterpretation=1"
/";                                     Include purely spatial clusters (y/n)"
/"IncludePurelySpatial=n"
/";                             Clusters to include (0=All, 1=Alive, 2=Range)"
/"IncludeClusters= 1"
/";                      Start range interval of window (YYYY/MM/DD,YYYY/MM/DD)"
/"IntervalStartRange=1900/1/1,1900/1/1"
/";                        End range interval of window (YYYY/MM/DD,YYYY/MM/DD)"
/"IntervalEndRange=1900/12/31,1900/12/31"


//"[Output Files]"
/"ResultsFile=" OUTFILENAME
/";                           Output most likely clusters in ASCII format (y/n)"
/"MostLikelyClusterEachCentroidASCII=y"
/";                           Output most likely clusters in dBase format (y/n)"
/"MostLikelyClusterEachCentroidDBase=y"
/";                                   Report census areas in ASCII format (y/n)"
/"CensusAreasReportedClustersASCII=y"
/";                                   Report census areas in dBase format (y/n)"
/"CensusAreasReportedClustersDBase=y"
/";               Report Simulated Log Likelihoods Ratios in ASCII format (y/n)"
/"SaveSimLLRsASCII=n"
/";               Report Simulated Log Likelihoods Ratios in dBase format (y/n)"
/"SaveSimLLRsDBase=n"
/";                                 Report relative risks in ASCII format (y/n)"
/"IncludeRelativeRisksCensusAreasASCII=y"
/";                                 Report relative risks in dBase format (y/n)"
/"IncludeRelativeRisksCensusAreasDBase=y"
/";                  Criteria for reporting secondary clusters(0=NoGeoOverlap,"
/";    1=NoCentersInOther, 2=NoCentersInMostLikely, 3=NoCentersInLessLikely,"
/";                              4=NoPairsCentersEachOther, 5=NoRestrictions)"
/"CriteriaForReportingSecondaryClusters=1"
/";            Max reported geographic size (< max geographical cluster size%)"
/"MaxReportedGeoClusterSize=50.000000"
/";                        Use reported maximum geographical cluster size (y/n)"
/"UseReportOnlySmallerClusters=n"
```

```
//"[Elliptic Scan]"
/";                                          Number of ellipses (0-10)"
/"NumberOfEllipses=0"
/";                                          Ellipse shapes"
/"EllipseShapes="
/";                                          Ellipse angles"
/"EllipseAngles="
/";                                       Duczmal Compactness Correction (y/n)"
/"DuczmalCompactnessCorrection=n"


//"[Sequential Scan]"
/";                                          Sequential scan (y/n)"
/"SequentialScan=n"
/";                               Max iterations for sequential scan (0-32000)"
/"SequentialScanMaxIterations=0"
/";                              Max p-Value for sequential scan (0.000-1.000)"
/"SequentialScanMaxPValue=0.000000"
/" "
/"[Advanced Features]"
/";                                          Validate parameters (y/n)"
/"ValidateParameters=y"
/";                                     Isotonic Scan (0=Standard, 1=Monotone)"
/"IsotonicScan=0"
/";                                    p-Values for 2 Prespecified LLR's (y/n)"
/"PValues2PrespecifiedLLRs=n"
/"LLR1=0.000000"
/"LLR2=0.000000"
/"EarlySimulationTermination=y"
/";    Simulated data methods (Null Randomization=0, HA Randomization=1, File
Import=2)"
/"SimulatedDataMethodType=0"
/";                         Simulated date input file name (with File Import=2)"
/"SimulatedDataInputFilename="
/";                         Use adjustments by known relative risks file? (y/n)"
/"UseAdjustmentsByRRFile=n"
/";      Adjustments by known relative risks file name (with HA
Randomization=1 or ...)"
/"AdjustmentsByKnownRelativeRisksFilename="
/";                                         Print simulated data to file (y/n)"
/"PrintSimulatedDataToFile=n"
/";                                          Simulated data output file name"
/"SimulatedDataOutputFilename="
/"MaxCirclePopulationFile=";
%Mend TractParam;


%macro callSatscan;
%macro dummy; %mend dummy;
/* Create the batch file that invokes SaTScan */
data _null_;
      /* noxwait option closes the SaTScan screen when run is finished */
      options noxwait mprint;
      file "C:\SaTScan9\Spatial.bat";
```

```
      string='"'||"C:\SaTScan9\SatScanBatch.exe"||'"
"'||"&&PARAM.param.txt"||'"';
      put string;
run;

/* Run SaTScan batch file */
x "C:\SaTScan9\Spatial.bat"; Run;

/* Read in the SaTScan output */

/* Col file: One row per identified cluster with following fields: */
/*    Cluster number, central census tract, X & Y coordinates, radius(ft), */
/*    cluster start & end dates, number of census tracts involved, test */
/*    statistic, p-value, recurrence interval, observed cases, expected */
/*    cases, observed/expected ratio */
PROC IMPORT OUT= WORK.OutCol
            DATAFILE=
"&OUTPUT\SpatialOut_&&disease_code&i..&&maxTemp&i.._&&agegroup&i...col.dbf"
                DBMS=DBF REPLACE;
     GETDELETED=NO;
RUN;

/* GIS file: One row per census tract with following fields */
/*    Census tract, cluster number, p-value, recurrence interval, */
/*    observed cases in cluster, expected cases in cluster, */
/*    observed/expected ratio in cluster, observed cases in census tract, */
/*    expected cases in census tract, observed/expected ratio in census tract
*/
PROC IMPORT OUT= WORK.OUTGIS
            DATAFILE=
"&OUTPUT\SpatialOut_&&disease_code&i..&&maxTemp&i.._&&agegroup&i...gis.dbf"
            DBMS=DBF REPLACE;
     GETDELETED=NO;
RUN;
%mend callSatscan;


/* This macro formats SaTScan output, appends all cluster info to archive, */
/*  and determines if there are any new clusters or events */
%macro switch;
%macro dummy; %mend dummy;
data clusterinfo;
      set outcol;
      length disease_code $45 agegroup $15;
      cluster=compress(cluster);
      end_date=compress(end_date);
      start_date=compress(start_date);
      radiusMile = radius/5280; format radiusMile 6.2;
/* The start_date and end_date variables in SaTScan output are string */
/*    variables in form YYYY/MM/DD, which SAS cannot read. */
      Clusterstartdate=mdy(input(scan(start_date,2,"/"),2.),
        input(scan(start_date,3,"/"),2.),input(scan(start_date,1,"/"),4.));
      format clusterstartdate mmddyy10.;
      Clusterenddate=mdy(input(scan(end_date,2,"/"),2.),
        input(scan(end_date,3,"/"),2.),input(scan(end_date,1,"/"),4.));
      format clusterenddate mmddyy10.;
```

```
      NumClusterDays=(clusterenddate-clusterstartdate)+1;
      runDate=&todaynum; format rundate mmddyy10.;
      disease_code ="&&disease_code&i";
      agegroup ="&&agegroup&i";
      fipsCount=substr(loc_id,3,3);
      if fipsCount='085' then Centroidboro='STATEN ISLAND';
      if fipsCount='005' then Centroidboro='BRONX';
      if fipsCount='081' then Centroidboro='QUEENS';
      if fipsCount='047' then Centroidboro='BROOKLYN';
      if fipsCount='061' then Centroidboro='MANHATTAN';
      drop start_date end_date fipscount;
run;

/* Keep only clusters over predetermined recurrence interval threshold. */
/* If all id'd clusters are below this threshold then keep the cluster */
/* with the lowest p-value (least likely cluster) */
proc sort data=clusterinfo; by cluster p_value; run;
data clusterinfo2;
      set clusterinfo;
      if _n_=1 or recurr_int>=&&recurrence&i;
run;

/* Keep fields to append to clusterhistory and satscanlinelist datasets, */
/* and for use in output if needed */
proc sql;
      create table ClusterHistory as
      select disease_code
            ,agegroup
            ,centroidBoro
            ,cluster
            ,x
            ,y
            ,radiusMile format=6.2
            ,clusterstartdate
            ,clusterEndDate
            ,numClusterdays
            ,runDate
            ,recurr_int
            ,p_value format 6.2
      /* populate analysis parameter fields */
            ,&&recurrence&i as Recurrence
            ,&&maxGeog&i as MaxGeog
            ,&&maxTemp&i as MaxTemp
            ,&&monteCarlo&i as MonteCarlo
            ,&&baseline&i as Baseline
            ,0 as NumConfProbSusp
      from clusterInfo2;
quit;

/* Append saved cluster details to clusterhistory file */
/*    On first run you will either need to copy the clusterhistory file to */
/* the support library instead of running proc append, or have a prepared */
/* file with test rows in the support library, which you can be deleted */
/* when no longer needed */
proc append base=support.Clusterhistory data=ClusterHistory;run;
proc sort data=support.Clusterhistory out=support.Clusterhistory nodupkey;
```

```
      by disease_code agegroup maxtemp rundate;run;

/* Select unique census tracts in clusters above the recurrence */
/* interval threshold */
proc sort data=outgis; by loc_id recurr_int; run;
data map_case;
      set outGIS;
      if recurr_int>=&&recurrence&i;
      x=lag(loc_id);
      if x=loc_id then delete;
      tract=loc_id;
      keep tract p_value recurr_int;
run;

/* If there are any census tracts in this count, then macros will run to */
/* set up data for output. Output is generated if &numnew >0 */
%global switch;
proc sql noprint;
      select count(*)
      into :Switch
      from map_case
      where recurr_int>=&&recurrence&i;
quit;
%if &switch = 0 %then %return;
%mend switch;

/* Set up the data for the map with this macro */
%macro CHOROPLETH_setup;
%macro dummy; %mend dummy;

/* read in NYC census tracts from shapefile */
proc mapImport
DATAFILE='S:\BCD\COMDISshared\Analyst_of_the_week\Maven\SaTScan\SupportingFil
es\census_tract_SF1SF32K_OEM_2010.shp'
      out=CityCensusTracts;
run;
data map_tract;
  set CityCensusTracts
    (keep=x y segment acres borocode nhoodcode puma shape_area shape_len
sq_miles tract); run;
proc sort data=map_tract;by tract;run;
proc sort data=map_case; by tract;run;

/* If a census tract is in more than one cluster, retain affiliation with */
/* least likely of associated clusters */
proc sort data=outgis; by loc_id cluster; run;
data outGIS2;
      set outgis;
      by loc_id;
      if first.loc_id=1;
run;

/* Merge all NYC census tracts with associated cluster and */
/*  recurrence interval */
proc sort data=citycensustracts; by tract segment; run;
proc sort data=map_tract; by tract; run;
```

```
proc sql;
      create table RemoveRecurrence1 as
      select ct.*
            ,og.cluster
            ,og.recurr_int
      from citycensustracts as ct
      left join outgis2 as og
      on ct.tract = og.loc_id;
quit;

/* If census tract is not part of a cluster, label as cluster 99 */
/*  (for mapping) */
data RemoveRecurrence2;
      set RemoveRecurrence1;
      if recurr_int<&&recurrence&i then cluster=99;
      if x ^=.;
run;

/* "proc gremove" eliminates borders between census tracts sharing */
/*  cluster affiliation */
proc sort data=RemoveRecurrence2 out=RemoveRecurrence3; by cluster tract;
run;
proc gremove data=RemoveRecurrence3 out=RemoveRecurrence4;
      by cluster;
      id tract;
run;
data RemoveRecurrence5;
      set RemoveRecurrence4;
      ClusterMap=0;
      if cluster ^= 99 then Clustermap=cluster;
run;

/* Macros for assigning fill colors and patterns for mapping */
%global realClusterNum;
%global clusternum;
proc sql noprint;
      select (max(clustermap)), (max(clustermap))+1
      into: RealClusterNum, :clusternum
      from RemoveRecurrence5;
quit;
%mend choropleth_setup;


/* If there is a cluster in the output, set up the data for the */
/* cluster summary page with this macro */
%macro linelist_setup;
%macro dummy; %mend dummy;

/* select individuals in a significant census tract */
proc sql;
      create table Satscan3 as
      select distinct s.*
      from satscan1 as s
            ,map_Case as mc
      where mc.tract = s.censusTract and s.active='yes';
quit;
```

```
/* join individuals on census tract to shapefile variables */
proc sql;
      create table ClusterToCensus as
      select s3.*, og.cluster
      from outGIs2 as og
      left join satscan3 as s3
      on og.loc_id = s3.censusTract
      order by event_id, cluster;
quit;

/* If in more than one cluster, retain affiliation with */
/* least likely of associated clusters */
data clustertocensus2;
      set clustertocensus;
      by event_id;
      if first.event_id=1;
run;

/* Add cluster start and end dates */
proc sql;
      create table linelist1 as
      select c2c2.*
            ,ci.clusterstartdate
            ,ci.clusterenddate
      from clustertocensus2 as c2c2
            ,clusterInfo as ci
      where c2c2.cluster=ci.cluster
      and event_id ^=''
      order by event_id, cluster;
quit;

/* Exclude event with "Not A Case" disease status or event date */
/* outside the cluster window */
data linelist2;
      set linelist1;
      by event_id cluster;
      lagEvent=lag(event_id);
      if lagEvent=event_id then delete;
      if disease_status not in ('NOT_A_CASE');
      NumClusterDays=(clusterenddate-clusterstartdate)+1;
      if event_date >= ClusterStartDate;
      if event_date <= ClusterEndDate;
      attrib InvestStat length=$20.;
      InvestStat=investigation_status;
      runDate=&todaynum; format rundate mmddyy10.;
      MaxTemp=&&maxtemp&i;
run;

/* Make a new dataset to retain the current cluster affiliation */
proc sql;
create table current_cluster as
select distinct event_id,
                        cluster
      from linelist2
      order by event_id;
```

```
quit;

/* Merge to compare current events with events already in */
/*  SaTScan linelist archive */

/* Linelist2: Cluster events that are new or exist in historical SaTScan */
/*    linelist to print case linelist in output */
/* existing_&&disease_code&i.._&&maxtemp&i.._&&agegroup&i..: Cluster */
/*    events in historical satscan linelist to use in defining a signal as */
/*    new or ongoing */
/*  On first run you will either need to copy the satscanlinelist file to */
/*    the support library instead of running proc append, or have a */
/*    prepared file with test rows in the support library, which can be */
/*    deleted when no longer needed */

proc sort data=linelist2;by event_id disease_code maxtemp agegroup;run;
proc sort data=support.satscanlinelist;by event_id disease_code maxtemp
agegroup;run;
data linelist2 existing_&&disease_code&i.._&&maxtemp&i.._&&agegroup&i..;
      merge linelist2 (in=a) support.satscanlinelist (in=b);
      by event_id;
      if a;
      if a & ~b then New = "*";
      if a then output linelist2;
      if a & b then output
existing_&&disease_code&i.._&&maxtemp&i.._&&agegroup&i..;
run;

/* Here we look for events with addresses in the geographic area */
/* of the cluster who are not in the cluster to generate a */
/* supplemental linelist */

/* Pull all addresses in Maven address history table from */
/* cluster-associated census tracts. These addresses will be used to */
/* output a linelist of cases whose primary residential addresses were */
/* outside the temporal and spatial extent of the cluster, but had other */
/* addresses (e.g., secondary home or work addresses) that fell within the */
/* cluster. Although these alternative addresses do not contribute to */
/* signal generation, this output could help investigators evaluate */
/* additional cases that might be related. */
proc sql;
      create table Satscan4 as
      select distinct s.*
      from mavensas.maven_addresshistory as s
            ,map_Case as mc
      where mc.tract = s.censustract
      order by party_id, reportdate;
quit;
data satscan4;
      set satscan4;
      by party_id reportdate;
      if first.party_id;
run;

/* Merge with analyst of the week (AOW) events table on Party ID, which */
/* is unique to person, not disease event. Remove legionellosis (LEG) */
```

```
/* cases from case file to account for past outbreaks in study period */

data satscan5;
      merge maven.dd_aow_events (keep=party_id event_id disease_status
      disease_code    event_date uhf boro gender investigation_status zip
      age) satscan4 (in=b);
      by party_id;
      if b;
      event_date = datepart(event_date);
      format event_date mmddyy10.;
      if disease_code="LEG" and disease_status="UNRESOLVED" then delete;
      /* Removing LEG cases from period of South Bronx 2015 outbreak */
            if disease_code="LEG" and event_date>='08JUL2015'd and
            event_date<='03AUG2015'd then delete;
      /* Removing LEG cases in nursing home nosocomial outbreak */
            if disease_code="LEG" and event_id in("100308152", "100308722",
            "100310829", "100310830", "100310831", "100311061", "100313746")
            then delete;
      /* Removing LEG cases from period of outbreak A */
            if disease_code="LEG" and event_date>='25APR2015'd and `
            event_date<='15MAY2015'd then delete;
      /* Removing LEG cases from period of outbreak B */
            if disease_code="LEG" and event_date>='04NOV2014'd and
            event_date<='31DEC2014'd then delete;
      /* Removing LEG cases from period of outbreak C */
            if disease_code="LEG" and event_date>='14SEP2015'd and
            event_date<='21SEP2015'd then delete;
      /* Removing Shiga toxin-producing E. coli (STEC) cases with positive */
      /* BioFire test only */
            if disease_code="STEC" and event_id in(&stec_biofire) then
delete;
run;

/* Add cluster ID by census tract */
proc sql;
      create table ClusterToCensus as
      select s3.*, og.cluster
      from outGIs2 as og
      left join satscan5 as s3
      on og.loc_id = s3.censusTract
      order by event_id, cluster;
quit;
data clustertocensus2;
      set clustertocensus;
      by event_id;
      if first.event_id=1;
run;

/* Add cluster start and end dates */
proc sql;
      create table linelist5 as
      select c2c2.*
            ,ci.clusterstartdate
            ,ci.clusterenddate
      from clustertocensus2 as c2c2
            ,clusterInfo as ci
```

```
      where c2c2.cluster=ci.cluster
      and event_id ^=''
      order by event_id, cluster;
quit;

/* Keep one row per disease event meeting spatio-temporal cluster */
/* definition */
/* Exclude "Not A Case" */
data linelist6;
      set linelist5;
      by event_id cluster;
      lagEvent=lag(event_id);
      if lagEvent=event_id then delete;
      if disease_status not in ('NOT_A_CASE');
      if disease_code = "&&disease_code&i";
      NumClusterDays=(clusterenddate-clusterstartdate)+1;
      if event_date >= ClusterStartDate & reportdate >= ClusterStartDate &
      event_date <= ClusterEndDate & reportdate <= ClusterEndDate;
      attrib InvestStat length=$20.;
      InvestStat=investigation_status;
      runDate=date(); format rundate mmddyy10.;
run;

/* Exclude events already in primary linelist */
proc sort data = linelist6; by event_id; run;
proc sort data = linelist2; by event_id; run;
data linelist7;
      merge linelist6 (in=a) linelist2 (in=b keep=event_id);
      by event_id;
      if a & ~b then output;
run;

/* This is where we look for additional relevant addresses for individuals */
/*  already in the cluster to generate a supplemental linelist */
proc sql;
      create table cluster_partyids as
      select distinct ll2.event_id,
                              e.party_id,
                              ll2.x_coord,
                              ll2.y_coord,
                              ll2.ClusterStartDate,
                              ll2.ClusterEndDate
      from linelist2 as ll2
            ,maven.dd_aow_events as e
      where ll2.event_id = e.event_id
      order by party_id;
quit;

/* Create macro variable with party IDs of events in cluster */
proc sql;
select distinct quote(party_id)
      into :cluster_partyids separated by ", "
      from cluster_partyids;
quit;

/* Pull all addresses from Maven address history table for people in */
```

```
/* cluster */
/* Format fields to match existing address fields */
data all_cluster_addresses1;
      set maven.dd_address_history;
      where party_id in(&cluster_partyids);
            rename CUSTOM_FIELD1=x_coord2;
            rename CUSTOM_FIELD2=y_coord2;
            format lat $50.;
            format long $50.;
            lat=strip(put(latitude,best12.));
            long=strip(put(longitude,best12.));
            rename street1=street_1;
            rename street2=street_2;
            format boro $50.;
            if county="Kings County (Brooklyn)" then boro="BROOKLYN";
            if county="Queens County (Queens)" then boro="QUEENS";
            if county="Richmond County (Staten Island)" then boro="STATEN
ISLAND";
            if county="Bronx County (Bronx)" then boro="BRONX";
            if county="New York County (Manhattan)" then boro="MANHATTAN";
            rename POSTAL_CODE_PREFIX=zip;
            rename CUSTOM_FIELD6=uhf;
            CENSUS_TRACT_2000_NUM=tract*1;
            rename tract=CENSUS_TRACT_2000;
            rename custom_field3=CENSUS_TRACT_2010;
            rename custom_field4=BIN;
            rename custom_field5=community_district;
            rename custom_field7=rpad_building_class;
run;

/* Keep relevant fields for successfully geocoded addresses */
data all_cluster_addresses2 (keep=party_id x_coord2 y_coord2 lat long
type_disp street_1 street_2 city state boro zip country uhf bin block
community_district rpad_building_class CensusTract address_startdate
address_enddate);
      set all_cluster_addresses1;
      /* format census tract */
            if census_tract_2000 ^='';
      StateNum=strip('36');
      /* Change Boro for Marble Hill addresses */
            if index(census_tract_2000,'309')~=0 and boro='BRONX' then
Boro='MANHATTAN';
      /* Rikers Island men's jails */
            if census_tract_2000 in ('   1','000100') and zip='11370' then
Boro='BRONX';
            if boro = 'BRONX' then BoroCode=strip('005');
            if boro = 'QUEENS' then BoroCode=strip('081');
            if boro = 'BROOKLYN' then BoroCode=strip('047');
            if boro = 'MANHATTAN' then boroCode=strip('061');
            if boro = 'STATEN ISLAND' then borocode=strip('085');
            CensusTract =
substr(compress(stateNum||BoroCode||census_tract_2000),1,11);
            format address_enddate mmddyy10.;
            address_enddate=datepart(end_date);
            format address_startdate mmddyy10.;
            address_startdate=datepart(start_date);
```

```
            if address_enddate='01JAN2030'd then address_enddate=&todaynum;
            drop census_tract_2000 borocode statenum;
run;

/* Keep only addresses in significant census tracts */
proc sql;
create table relevant_addresses as
      select aca.*
      from all_cluster_addresses2 as aca,
             map_Case as mc
      where aca.censustract=mc.tract;
quit;

/* Keep only addresses that are unique from address used in analysis */
/*  and were active during the cluster */
proc sort data=relevant_addresses;by party_id;run;
data other_relevant_addresses;
merge relevant_addresses (in=a) cluster_partyids (in=b);
      by party_id;
      if x_coord=x_coord2 and y_coord=y_coord2 then delete;
      if (address_startdate<=ClusterEndDate and
address_enddate>=ClusterEndDate) or
       (address_startdate<=ClusterStartDate and
address_enddate>=ClusterStartDate) or
       (address_startdate<=ClusterStartDate and
address_enddate>=ClusterEndDate) or
       (address_startdate>=ClusterStartDate and
      address_enddate<=ClusterEndDate)
      then output;
run;

%mend linelist_setup;


/* Macro to determine if any new events:  */
/*    1. Form a new cluster   or    2. Were added to an ongoing cluster */
%macro NewIndividuals;
%macro dummy; %mend dummy;

proc sql;
      create table LineListIndividuals as
      select distinct event_id
            ,disease_code
            ,agegroup
            ,event_date
            ,cluster
            ,rundate
            ,maxtemp
      from linelist2
      order by event_id, rundate;
quit;

/* Count number of confirmed, probable, and suspect cases */
proc sql;
      select count(*) as count
      into :count
```

```
      from linelist2
      where disease_status in ("CONFIRMED","PROBABLE","SUSPECT");
quit;
%put &count;

/* Add confirmed, probable, and suspect count to cluster history */
data clusterhistory;
      set clusterhistory;
      NumConfProbSusp = &count.;
run;


/* Add confirmed, probable, and suspect count to archived cluster */
/* history dataset */
proc sort data = clusterhistory;
      by disease_code agegroup maxtemp rundate cluster;
run;
proc sort data = support.clusterhistory;
      by disease_code agegroup maxtemp rundate cluster;
run;
data support.clusterhistory;
      merge support.clusterhistory (in=a) clusterhistory (in=b);
      by disease_code agegroup maxtemp rundate cluster;
      if a;
run;

/* Keep events new to SaTScan linelist by disease and analysis parameters */
proc sql;
      create table NewIndividuals as
      select lli.*
      from linelistIndividuals as lli
      left join support.satscanlinelist as s
      on lli.event_id=s.event_id and lli.disease_code=s.disease_code
            and lli.agegroup=s.agegroup and lli.maxtemp=s.maxtemp
      having s.event_id ='';
quit;

/* Count number of rows in dataset of new individuals. */
/* This determines whether macros to produce output will be run */
/* This will only happen if &switch > 0 */
%let dsid=%sysfunc(open(NewIndividuals));
%global numnew;
%let numnew=%sysfunc(attrn(&dsid,nobs));
%let rc=%sysfunc(close(&dsid));
%if &NumNew=0 %then %return;
%mend NewIndividuals;

/* Append new individuals to historic SaTScan Linelist */
%macro AddToList;
%macro dummy; %mend dummy;
proc sort data=NewIndividuals; by event_id disease_code agegroup maxtemp;run;
proc append base=support.SatScanLineList data=NewIndividuals;run;
proc sort data=support.SatScanLineList out=support.SatScanLineList nodupkey;
      by event_id disease_code agegroup maxtemp;
run;
%mend AddtoList;
```

```
/* Macro to generate choropleth map of significant clusters over */
/* NYC outline base map */
%macro MakeChoropleth;
%macro dummy; %mend dummy;
data annotate;
      set LineList2 ;
      length style color $ 1 text $ 20;
      retain xsys ysys '2' hsys '3' when 'a';
      /*Add points for each address in cluster */
      function='label'; style='arial'; text='*'; size=1.5; color = "CYAN";
run;

/* Open word file for output */
ods listing close;
ods rtf body =
"S:\BCD\COMDISshared\Analyst_of_the_week\Maven\SaTScan\archive\&today\satscan
_&&Disease_Code&i.._&&agegroup&i.._&&maxTemp&i...rtf";
ods rtf file
="S:\BCD\COMDISshared\Analyst_of_the_week\Maven\SaTScan\archive\&today\satsca
n_&&Disease_Code&i.._&&agegroup&i.._&&maxTemp&i...rtf";
ods rtf style=Meadow;

/* Set options and title statements */
goptions reset=goptions device=png300 target=png300 ftext='Calibri'
ftitle='Calibri/bold' htitle=2 xmax=9 in ymax=7 in;
title1 height=1.5 "&&disease_code&i clusters with event dates through
&Simendformat";
title2 height=1.3 "Confirmed, probable, suspected and pending cases";

/* Set gradient of 10 possible cluster color patterns, */
/* plus one blank fill pattern for census tracts not affiliated */
/* with a cluster */
pattern1 value=msolid color=CXfc8d59;
pattern2 value=msolid color=CXd6ad4b;
pattern3 value=msolid color=CXfee08b;
pattern4 value=msolid color=CXfdfd65;
pattern5 value=msolid color=CXffffbf;
pattern6 value=msolid color=CXe6f598;
pattern7 value=msolid color=CXb7f291;
pattern8 value=msolid color=CX99d594;
pattern9 value=msolid color=CX51d06d;
pattern10 value=msolid color=CX4b9cd8;
pattern11 value=msolid color=CXECEDEC;

/* Set cluster color pattern based on number of clusters represented */
/* on map */
data _null_;
%if &CLusterNum=2
      %then %do;
      pattern2 value=msolid color=CXECEDEC;
      %end;
%if &CLusterNum=3
      %then %do;
      pattern3 value=msolid color=CXECEDEC;
      %end;
```

```
%if &CLusterNum=4
      %then %do;
      pattern4 value=msolid color=CXECEDEC;
      %end;
%if &CLusterNum=5
      %then %do;
      pattern5 value=msolid color=CXECEDEC;
      %end;
%if &CLusterNum=6
      %then %do;
      pattern6 value=msolid color=CXECEDEC;
      %end;
%if &CLusterNum=7
      %then %do;
      pattern7 value=msolid color=CXECEDEC;
      %end;
%if &CLusterNum=8
      %then %do;
      pattern8 value=msolid color=CXECEDEC;
      %end;
%if &CLusterNum=9
      %then %do;
      pattern9 value=msolid color=CXECEDEC;
      %end;
%if &CLusterNum=10
      %then %do;
      pattern10 value=msolid color=CXECEDEC;
      %end;
run;

/* Set footnotes */
OPTIONS NOCENTER  nonumber  ls=140 ps=51 nodate;
footnote1 font='Arial' justify=left height=1 'Statistic: Prospective space-
time permutation scan statistic';
footnote2 font='Arial' justify=left height=1 'Spatial resolution: census 2000
tracts';
footnote3 font='Arial' justify=left height=1 "Baseline period: &&baseline&i
days";
footnote4 font='Arial' justify=left height=1 "Maximum temporal cluster size
(days): &&maxtemp&i";
footnote5 font='Arial' justify=left height=1 "Maximum spatial cluster size (%
of cases): &&maxgeog&i";
footnote6 font='Arial' justify=left height=1 "Number of Monte Carlo
simulations: &&montecarlo&i";
footnote7 font='Arial' justify=left height=1 'Criteria for reporting
secondary clusters: no cluster center in other clusters';
footnote8 font='Arial' justify=left height=1 "Only recurrence interval >=
&&recurrence&i days are shown";
footnote9 font='Arial' justify=left height=1 'Adjusted for space by day-of-
week interaction';

/* Generate map in output */
proc gmap map=RemoveRecurrence5 data=RemoveRecurrence5 anno=annotate;
      id cluster;
      choro cluster/ levels=&clusternum
      coutline=gray
```

```
            CDEFAULT = white
            cempty=blue
            cempty=black
            legend=legend1;
run;
quit;
%mend MakeChoropleth;


%macro MakeClusterLineList;
%macro dummy; %mend dummy;

/* Set options and title statements */
title1  "Cluster information";
title2 height=1.5 "Cluster #1 is the most likely cluster, i.e. the cluster
least likely to be due by chance";

footnote1 font='Arial' height=2 '*Recurrence interval represents the expected
length of follow-up required to see one cluster ';
footnote2 font='Arial' height=2 '           at least as unusual as the
observed cluster by chance';

/* Format select variables for output */
proc sql;
      create table clusterinfoGraph as
      select distinct cluster
            ,clusterstartdate
            ,clusterenddate
            ,numclusterdays
            ,radiusMile format = 5.2
            ,ode format=4.2
            ,recurr_int
      from clusterinfo
      where recurr_int >= &&recurrence&i;
quit;

/* Output linelist of cluster information to word document */
/*  Row color corresponds to color of cluster on map */
proc report data=clusterinfoGraph nowd;
column cluster clusterstartdate clusterenddate numclusterdays radiusMile ode
recurr_int;
      define cluster / order 'Cluster' width=1;
      define clusterstartdate /'Start date' width = 18;
      define clusterenddate/ 'End date' width=18;
      define NumClusterDays/'# days in cluster' width=5;
      define radiusMile/'Radius (miles)' width=5 ;
      define ode / "Observed over Expected" width=4;
      define recurr_int / 'Recurrence interval (days)*' width=5;
      compute cluster;
      if cluster=1
      then call define (_ROW_, "style", "STYLE = [background=CXFC8D59]");
      if cluster=2
      then call define (_ROW_, "style", "STYLE = [background=CXd6ad4b]");
      if cluster=3
      then call define (_ROW_, "style", "STYLE = [background=CXfee08b]");
      if cluster=4
```

```
      then call define (_ROW_, "style", "STYLE = [background=CXfdfd65]");
      if cluster=5
      then call define (_ROW_, "style", "STYLE = [background=CXffffbf]");
      if cluster=6
      then call define (_ROW_, "style", "STYLE = [background=CXe6f598]");
      if cluster=7
      then call define (_ROW_, "style", "STYLE = [background=CXb7f291]");
      if cluster=8
      then call define (_ROW_, "style", "STYLE = [background=CX99d594]");
      if cluster=9
      then call define (_ROW_, "style", "STYLE = [background=CX51d06d]");
      if cluster=10
      then call define (_ROW_, "style", "STYLE = [background=CX4b9cd8]");
      ENDCOMP;
run;
%Mend MakeClusterlinelist;


%macro PersonLineList;
%macro dummy; %mend dummy;

/* Overwrite first cluster affiliation with current cluster affiliation */
data linelist2;
      merge linelist2 (in=a) current_cluster (in=b);
      by event_id;
      if a;
run;

/* Add United Hospital Fund (UHF) neighborhood name. UHF neighborhoods are */
/*  aggregations of contiguous ZIP codes used to define NYC communities. */
proc sort data = linelist2; by uhf; run;
data linelist3;
      merge linelist2 (in=a) uhftable;
      by uhf;
      if a;
run;

/* Format for output */
proc sort data= linelist3; by event_id cluster; run;
data linelist4;
      set linelist3;
      attrib eventID length = $15.;
      eventID=event_ID;
      drop event_id;
      attrib DiseaseStat length=$20.;
      diseaseStat=disease_status;
      drop disease_status;
      attrib boroShort length=$5.;
      if boro = "BROOKLYN" then boroshort = "BK";
      if boro = "QUEENS" then boroshort = "QN";
      if boro = "STATEN ISLAND" then boroshort = "SI";
      if boro = "BRONX" then boroshort = "BX";
      if boro = "MANHATTAN" then boroshort = "MN";
      if gender='FEMALE' then gender ='F';
      if gender='MALE' then gender='M';
      drop boro;
```

```
run;

proc sort data=linelist3; by cluster uhf zip;run;

/* Set title and footnote statements */
title1 'Cluster information';
title2 'Individuals included in the cluster';
footnote1 '* Event ID did not appear on any previous report';
footnote2 ;

/* Output person linelist to word document */
proc report  data=linelist4 nowd ;
column cluster new eventid  diseasestat investStat event_date  age
      type street_1 boroshort zip uhfname censustract;
define cluster / order 'Cluster' width=1;
define New / 'New event*' width=1;
define diseasestat /'Disease status' width=5;
define investStat / 'Investigation status' width=4;
define eventid /'Event ID' width=8;
define event_date / 'Event date' format=mmddyy10. width=10;
define age / 'Age' width=3;
define type / 'Address type' width=8;
define street_1 /'Address' width=10;
define boroshort /'Boro'  width=10;
define zip /'Zip' width=5;
define uhfname /'UHF' width=10;
define censustract / 'Census tract' width=13;
compute cluster;
      if cluster=1
      then call define (_ROW_, "style", "STYLE = [background=CXFC8D59]");
      if cluster=2
      then call define (_ROW_, "style", "STYLE = [background=CXd6ad4b]");
      if cluster=3
      then call define (_ROW_, "style", "STYLE = [background=CXfee08b]");
      if cluster=4
      then call define (_ROW_, "style", "STYLE = [background=CXfdfd65]");
      if cluster=5
      then call define (_ROW_, "style", "STYLE = [background=CXffffbf]");
      if cluster=6
      then call define (_ROW_, "style", "STYLE = [background=CXe6f598]");
      if cluster=7
      then call define (_ROW_, "style", "STYLE = [background=CXb7f291]");
      if cluster=8
      then call define (_ROW_, "style", "STYLE = [background=CX99d594]");
      if cluster=9
      then call define (_ROW_, "style", "STYLE = [background=CX51d06d]");
      if cluster=10
      then call define (_ROW_, "style", "STYLE = [background=CX4b9cd8]");
endcomp;
run;




/* For invasive Group A Streptococcus (GAS) and */
```

```
/* invasive Streptococcus pneumonia (PNE) */
/* (both potential healthcare-associated infections), pull reporter and */
/* reporting facility information for inclusion in a supplemental */
/* linelist of facilities associated with individuals in cluster */
%if &disease_code="GAS" or &disease_code="PNE" %then %do;

/* Pull all disease reports */
data reports;
set maven.dd_aow_reports;
      where disease_code = &disease_code;
run;

/* Keep if event is in cluster */
proc sort data=linelist3;by event_id cluster;run;
proc sort data=reports;by event_id;run;
data linelist3_reports;
      retain event_id cluster hcf_name hcf_addressline1 hcf_addressline2
      hcf_city hcf_state hcf_zip hcf_phone reporter_facility_name;
      merge linelist3 (in=a) reports;
      by event_id;
      if a;
      keep event_id cluster hcf_name hcf_addressline1 hcf_addressline2
      hcf_city hcf_state hcf_zip hcf_phone reporter_facility_name;
run;

/* Keep one report per unique Health Care Facility name value per event */
proc sort data=linelist3_reports nodupkey;by cluster hcf_name event_id;run;

/* Set title and footnote statements */
title1 'Cluster information';
title2 'Reporting information for individuals included in the cluster';
footnote1 ;
footnote2 ;

/* Output reporting information to word document */
proc report  data=linelist3_reports nowd ;
column cluster event_id hcf_name hcf_phone hcf_addressline1 hcf_addressline2
hcf_city hcf_state hcf_zip reporter_facility_name;
define cluster /order 'Cluster' width=1;
define event_id /'Event ID' width=8;
define hcf_name /'Health Care Facility' width=12;
define hcf_phone /'HCF Contact' width=8;
define hcf_addressline1 /'HCF Address 1' width=13;
define hcf_addressline2 /'HCF Address 2' width=7;
define hcf_city /'HCF City' width=6;
define hcf_state /'HCF State' width=1;
define hcf_zip /'HCF Zip'  width=5;
define reporter_facility_name /'Reporter Facility' width=12;
compute cluster;
      if cluster=1
      then call define (_ROW_, "style", "STYLE = [background=CXFC8D59]");
      if cluster=2
      then call define (_ROW_, "style", "STYLE = [background=CXd6ad4b]");
      if cluster=3
      then call define (_ROW_, "style", "STYLE = [background=CXfee08b]");
      if cluster=4
```

```
      then call define (_ROW_, "style", "STYLE = [background=CXfdfd65]");
      if cluster=5
      then call define (_ROW_, "style", "STYLE = [background=CXffffbf]");
      if cluster=6
      then call define (_ROW_, "style", "STYLE = [background=CXe6f598]");
      if cluster=7
      then call define (_ROW_, "style", "STYLE = [background=CXb7f291]");
      if cluster=8
      then call define (_ROW_, "style", "STYLE = [background=CX99d594]");
      if cluster=9
      then call define (_ROW_, "style", "STYLE = [background=CX51d06d]");
      if cluster=10
      then call define (_ROW_, "style", "STYLE = [background=CX4b9cd8]");
endcomp;
run;
%end;

/* Count number of new cluster addresses to print in output */
%let new_cluster_add=%sysfunc(open(linelist7));
%let num_new_cluster_add=%sysfunc(attrn(&new_cluster_add,nobs));
%let end=%sysfunc(close(&new_cluster_add));

/* If any addresses to be output then run */
%if &num_new_cluster_add>0 %then %do;

/* Add UHF name */
proc sort data = linelist7; by uhf; run;
data linelist8;
      merge linelist7 (in=a) uhftable;
      by uhf;
      if a;
run;

/* Format for output */
proc sort data= linelist8; by event_id cluster; run;
data linelist9;
      set linelist8;
      attrib eventID length = $15.;
      eventID=event_ID;
      drop event_id;
      attrib DiseaseStat length=$20.;
      diseaseStat=disease_status;
      drop disease_status;
      attrib boroShort length=$5.;
      if boro = "BROOKLYN" then boroshort = "BK";
      if boro = "QUEENS" then boroshort = "QN";
      if boro = "STATEN ISLAND" then boroshort = "SI";
      if boro = "BRONX" then boroshort = "BX";
      if boro = "MANHATTAN" then boroshort = "MN";
      if gender='FEMALE' then gender ='F';
      if gender='MALE' then gender='M';
      drop boro;
run;

proc sort data=linelist9; by cluster uhf zip;run;
```

```
/* Set title and footnote statements */
title1 'Individuals not included in cluster by residential address at time of
report who have another home or work address in the cluster';
title2;
footnote1 ;
footnote2 ;

/* Output list of new addresses to word document */
proc report  data=linelist9 nowd ;
column cluster eventid  diseasestat investStat event_date  age
      street1 boroshort zip uhfname censustract;
define cluster / order 'Cluster' width=1;
define diseasestat /'Disease status' width=5;
define investStat / 'Investigation status' width=4;
define eventid /'Event ID' width=8;
define event_date / 'Event date' format=mmddyy10. width=10;
define age / 'Age' width=3;
define street1 /'Address' width=10;
define boroshort /'Boro'  width=10;
define zip /'Zip' width=5;
define uhfname /'UHF' width=10;
define censustract / 'Census tract' width=13;
compute cluster;
      if cluster=1
      then call define (_ROW_, "style", "STYLE = [background=CXFC8D59]");
      if cluster=2
      then call define (_ROW_, "style", "STYLE = [background=CXd6ad4b]");
      if cluster=3
      then call define (_ROW_, "style", "STYLE = [background=CXfee08b]");
      if cluster=4
      then call define (_ROW_, "style", "STYLE = [background=CXfdfd65]");
      if cluster=5
      then call define (_ROW_, "style", "STYLE = [background=CXffffbf]");
      if cluster=6
      then call define (_ROW_, "style", "STYLE = [background=CXe6f598]");
      if cluster=7
      then call define (_ROW_, "style", "STYLE = [background=CXb7f291]");
      if cluster=8
      then call define (_ROW_, "style", "STYLE = [background=CX99d594]");
      if cluster=9
      then call define (_ROW_, "style", "STYLE = [background=CX51d06d]");
      if cluster=10
      then call define (_ROW_, "style", "STYLE = [background=CX4b9cd8]");
endcomp;
run;
%end;

/* This is where we list additional relevant addresses for events */
/* in the cluster to generate a supplemental linelist */
/*  Count number of new cluster addresses to print in output */
%let rev_cluster_add=%sysfunc(open(other_relevant_addresses));
%let num_rev_cluster_add=%sysfunc(attrn(&rev_cluster_add,nobs));
%let end=%sysfunc(close(&rev_cluster_add));

/* If any additional relevant addresses to be output then run */
%if &num_rev_cluster_add>0 %then %do;
```

```
proc sort data=other_relevant_addresses nodupkey;by party_id x_coord
y_coord;run;

/* Keep if party ID is in cluster */
proc sql;
create table other_relevant_addresses2 as
      select a.event_id,
                  b.*
      from cluster_partyids as a,
            other_relevant_addresses as b
      where a.party_id=b.party_id
      order by event_id;
quit;

/* Keep if event ID is in cluster */
data other_relevant_addresses3;
      merge other_relevant_addresses2 (in=a) current_cluster (in=b);
      by event_id;
      if a and b;
run;

/* Add uhf name */
proc sort data = other_relevant_addresses3; by uhf; run;
data other_relevant_addresses_uhf;
      merge other_relevant_addresses3 (in=a) uhftable;
      by uhf;
      if a;
run;

/* Format for output */
proc sort data= other_relevant_addresses_uhf; by event_id cluster; run;
data other_relevant_addresses_recode;
      set other_relevant_addresses_uhf;
      attrib eventID length = $15.;
      eventID=event_ID;
      drop event_id;
      attrib Type length=$20.;
      Type=type_disp;
      drop type_disp;
      attrib boroShort length=$5.;
      if boro = "BROOKLYN" then boroshort = "BK";
      if boro = "QUEENS" then boroshort = "QN";
      if boro = "STATEN ISLAND" then boroshort = "SI";
      if boro = "BRONX" then boroshort = "BX";
      if boro = "MANHATTAN" then boroshort = "MN";
      if gender='FEMALE' then gender ='F';
      if gender='MALE' then gender='M';
      drop boro;
run;

/* Set title and footnote statements */
title1 'Individuals included in cluster by residential address at time of
report who also have another home or work address in the cluster area';
title2;
footnote1 ;
footnote2 ;
```

```
/* Output list of additional relevant addresses to word document */
proc report  data=other_relevant_addresses_recode nowd ;
column cluster eventid type address_enddate
      street_1 boroshort zip uhfname censustract;
define cluster / order 'Cluster' width=1;
define eventid /'Event ID' width=8;
define type / 'Address type' width=8;
define address_enddate / 'Address end date' format=mmddyy10. width=10;
define street_1 /'Address' width=10;
define boroshort /'Boro'  width=10;
define zip /'Zip' width=5;
define uhfname /'UHF' width=10;
define censustract / 'Census tract' width=13;
compute cluster;
      if cluster=1
      then call define (_ROW_, "style", "STYLE = [background=CXFC8D59]");
      if cluster=2
      then call define (_ROW_, "style", "STYLE = [background=CXd6ad4b]");
      if cluster=3
      then call define (_ROW_, "style", "STYLE = [background=CXfee08b]");
      if cluster=4
      then call define (_ROW_, "style", "STYLE = [background=CXfdfd65]");
      if cluster=5
      then call define (_ROW_, "style", "STYLE = [background=CXffffbf]");
      if cluster=6
      then call define (_ROW_, "style", "STYLE = [background=CXe6f598]");
      if cluster=7
      then call define (_ROW_, "style", "STYLE = [background=CXb7f291]");
      if cluster=8
      then call define (_ROW_, "style", "STYLE = [background=CX99d594]");
      if cluster=9
      then call define (_ROW_, "style", "STYLE = [background=CX51d06d]");
      if cluster=10
      then call define (_ROW_, "style", "STYLE = [background=CX4b9cd8]");
endcomp;
run;
%end;

/* Close word document */
ods rtf close;
ods listing;

%mend PersonLineList;
```

File 2: Analysis Program

```
/*************************************************************************/
/*    PROGRAM NAME: SaTScan_v5                                           */
/*    DATE CREATED: February 10, 2014                                    */
/*    LAST UPDATED: November 4, 2015                                     */
```

```
/*    PROGRAMMERS: Deborah Kapell                                        */
/*                 Eric Peterson                                         */
/*                                                                       */
/*    PURPOSE: Runs SaTScan analysis for all disease-analysis parameter */
/*    combinations using SaTScan v9.1.1 space-time permutation scan     */
/*    statistic                                                          */
/*                                                                       */
/************************************************************************/

/* Read in file with most of the macros needed for the analysis */
%include "\\nasprgshare220\share\DIS\BCD\COMDISshared\Analyst_of_the_week\
            Maven\SaTScan\Satscan_macros2.sas";

/* Create a file in the archive to store output from analysis */
options noxwait ;
x "cd S:\BCD\COMDISshared\Analyst_of_the_week\Maven\SaTScan\archive";
x "md &today";
x "exit";

/* delete today's lines from archived datasets if this is rerun on */
/* the same day */
proc sql;
delete * from support.satscanlinelist
where rundate=&todaynum;
quit;
proc sql;
delete * from support.clusterhistory
where rundate=&todaynum;
quit;


/* To exclude STEC with BioFire only - pull all BioFire STEC results */
/* from labs table */
data stec_labs (keep=event_id test_name);
set maven.dd_aow_labs;
      where disease_code="STEC";
      if index(upcase(RESULT_NAME),"NEGATIVE")>0 then delete;
      test_name=upcase(strip(test_name));
run;

/* sort and dedupe by test name to save only unique tests */
proc sort data=stec_labs nodupkey;by event_id test_name;run;

/* transpose to wide format */
proc transpose data=stec_labs out=stec_labs_wide;
      by event_id;
      var test_name;
run;

/* Concatenate unique tests */
data unique_stec_labs (keep = event_id unique_tests);
set stec_labs_wide;
unique_tests = catx('; ',of col:);
run;

/* Insert event IDs with only a BioFire result into macro variable */
```

```
proc sql;
      select distinct quote(event_id)
      into :stec_biofire separated by ", "
      from unique_stec_labs
      where unique_tests="BIOFIRE FILMARRAY GASTROINTESTINAL PANEL";
quit;

/*  This step imports data from a disease surveillance database, */
/*  determines what diseases require analysis, and produces case files in */
/* .txt format. Modifications may be required depending on the structure */
/* and content of the source data                                        */

/*  Pull events from AOW events table, excluding:           */
/*          1. Non-NYC addresses                            */
/*    `     2. Contacts and possible exposures              */
/*          3. Legionellosis (LEG) cases with unresolved case status    */
/* (LEG events are assigned as "Unresolved" when reported with an antibody */
/* test, a method requiring two results at least four weeks apart showing */
/* a four-fold change in titer to confirm a diagnosis. Such cases are not */
/* routinely investigated and are thus not of interest unless the first */
/* result is highly positive, a second antibody test shows a sufficient */
/* increase in titer, or a positive urine antigen test is reported, at */
/* which point the case status will be updated to "Confirmed." */
/* To account for this testing bias, we exclude unresolved LEG events */
/* from analysis.) */
/*          4. LEG cases from past outbreaks                */
/*          5. STEC cases with BioFire test only            */

data events;
      set maven.dd_aow_events
      (where=(((disease_code^="LEG" and disease_status not in ('CONTACT',
      'POSSIBLE_EXPOSURE'))
      or (disease_code="LEG" and disease_status not in ('CONTACT',
      'POSSIBLE_EXPOSURE', 'UNRESOLVED')))));
      event_date=datepart(event_date);
      format event_date mmddyy10.;
      CENSUS_TRACT_2000_NUM=input(CENSUS_TRACT_2000,??BEST12.);
/* Removing LEG cases from period of South Bronx 2015 outbreak */
      if disease_code="LEG" and event_date>='08JUL2015'd and
      event_date<='03AUG2015'd then delete;
/* Removing LEG cases in nursing home nosocomial outbreak */
      if disease_code="LEG" and event_id in("100308152", "100308722",
      "100310829", "100310830", "100310831", "100311061", "100313746") then
      delete;
/* Removing LEG cases from period of outbreak A*/
      if disease_code="LEG" and event_date>='25APR2015'd and
      event_date<='15MAY2015'd then delete;
/* Removing LEG cases from period of outbreak B */
      if disease_code="LEG" and event_date>='04NOV2014'd and
      event_date<='31DEC2014'd then delete;
/* Removing LEG cases from period of outbreak C */
      if disease_code="LEG" and event_date>='14SEP2015'd and
      event_date<='21SEP2015'd then delete;
/* Removing STEC cases with positive Biofire test only */
      if disease_code="STEC" and event_id in(&stec_biofire) then delete;
      type="Home";
```

```
run;

/* Pull geocoded secondary addresses for events that have a */
/*  non-geocodable address at time of report */
      proc sql;
      create table secondary_addresses as
      select distinct a.party_id,
                        b.*,
                        case
                              when type_disp="Home (Secondary)" then 1
                              when type_disp="Work" then 2
                              when type_disp="Work (Secondary)" then 3
                        end as hierarchy
      from events a, maven.DD_ADDRESS_HISTORY b
      where a.party_id=b.party_id and a.x_coord is missing and a.y_coord is
missing
            and (index(type_disp,"Work")>0 or type_disp="Home (Secondary)")
            and b.CUSTOM_FIELD1^=" " and b.CUSTOM_FIELD2^=" ";
      quit;
      proc sort data=secondary_addresses;by party_id hierarchy descending
end_date;run;
/* Keep most recent geocoded secondary address for each party. */
/* Prioritize, in order: secondary home, primary work, secondary work */
data secondary_address
      (keep=party_id x_coord y_coord lat long street_1 street_2 city state
      boro zip country uhf bin block community_district rpad_building_class
      CENSUS_TRACT_2000 CENSUS_TRACT_2000_NUM CENSUS_TRACT_2010 hierarchy);
set secondary_addresses;
      by party_id hierarchy descending end_date;
      if first.party_id;
      rename CUSTOM_FIELD1=x_coord;
      rename CUSTOM_FIELD2=y_coord;
      format lat $50.;
      format long $50.;
      lat=strip(put(latitude,best12.));
      long=strip(put(longitude,best12.));
      rename street1=street_1;
      rename street2=street_2;
      format boro $50.;
      if county="Kings County (Brooklyn)" then boro="BROOKLYN";
      if county="Queens County (Queens)" then boro="QUEENS";
      if county="Richmond County (Staten Island)" then boro="STATEN ISLAND";
      if county="Bronx County (Bronx)" then boro="BRONX";
      if county="New York County (Manhattan)" then boro="MANHATTAN";
      rename POSTAL_CODE_PREFIX=zip;
      rename CUSTOM_FIELD6=uhf;
      CENSUS_TRACT_2000_NUM=input(tract,??BEST12.);
      rename tract=CENSUS_TRACT_2000;
      rename custom_field3=CENSUS_TRACT_2010;
      rename custom_field4=BIN;
      rename custom_field5=community_district;
      rename custom_field7=rpad_building_class;
run;

/* Count number of secondary addresses to be added to events dataset */
%let sec_add=%sysfunc(open(secondary_address));
```

```
%let num_secondary_address=%sysfunc(attrn(&sec_add,nobs));
%let end=%sysfunc(close(&sec_add));

/* Macro to replace non-geocodable address at time of report with */
/*  geocoded secondary address elements if any exist */
%macro add_secondary_address (data1,data2);
%macro dummy; %mend dummy;
%if &num_secondary_address > 0 %then %do;
      proc sort data=&data1;by party_id;run;

/* Add geocoded secondary address fields to events in place of */
/*  address at time of report that did not geocode */
data &data1 (drop=hierarchy);
      merge &data1 (in=a) &data2 (in=b);
            by party_id;
            if boro not in ('OUTSIDE NYC' 'NA');
            if a and b and hierarchy in(2,3) then type = "Work";
      run;
%end;
%mend add_secondary_address;

/* Run macro with specific datasets */
%add_secondary_address(events,secondary_address);

/* Create a dataset of diseases with analysis parameters that have */
/*  at least one event within the maximum temporal window */
/* This will be used to create macro variables for performing separate */
/*  SaTScan analyses by unique disease-analysis parameter combinations */
proc sql;
      create table diseaseListCurrent as
      select distinct m.disease_code
            ,s.recurrence
            ,s.maxtemp
            ,s.maxgeog
            ,s.baseline
            ,s.montecarlo
            ,s.lagtime
            ,s.agegroup
      from events as m
            ,support.diseaselist as s
      where m.disease_code = s.disease_code
      and event_date >=intnx('day',&todaynum,-((maxtemp-1)+lagtime)) and
                  event_date <=intnx('day',&todaynum,-lagtime)
      group by m.disease_code, s.recurrence, s.maxtemp, s.maxgeog,
      s.baseline,  s.montecarlo, s.lagtime, s.agegroup having
      max(CENSUS_TRACT_2000_NUM)^=.
      order by disease_code;
quit;

/* create variables to loop through for analyses */
data _NULL_;
      set diseaseListCurrent;
      by disease_code maxTemp agegroup recurrence;
      if first.disease_code | first.maxTemp | first.agegroup then do;
            i+1;
```

```
            call symputx
('disease_code'||left(put(i,5.)),strip(disease_code));
            call symputx ('lagtime'||left(put(i,2.)),lagtime);
            call symputx ('recurrence'||left(put(i,2.)),recurrence);
            call symputx ('endloop' ,left(put(i,3.)));
            call symputx ('maxTemp'||left(put(i,2.)),maxTemp);
            call symputx ('maxGeog'||left(put(i,2.)),maxGeog);
            call symputx ('monteCarlo'||left(put(i,2.)),monteCarlo);
            call symputx ('Baseline'||left(put(i,2.)),baseline);
            call symputx ('agegroup'||left(put(i,2.)),agegroup);
      end;
run;

/* Run SaTScan analyses, looping through unique disease-analysis */
/* parameter iterations */
%macro RunProgram;
%macro dummy; %mend dummy;
%do i=1 %to &endloop;
data _null_;
/* START AND END DATE OF ANALYSIS */
/* First date analyzed in simulation */
      %global simstart;
      simstart=&todaynum-(&&baseline&i+&&lagtime&i);
      call symputx('simstart',simstart);
      call symputx('SimStartFormat',put(simstart,date7.));
/* Last date analyzed in simulation */
      %global simend;
      simend  =&todaynum- &&lagtime&i;
      call symputx('simend',simend);
      call symputx('SimEndFormat',put(simend,worddate18.));
/* First date of active window */
      %global simActive;
      simActive = &simend- (&&maxtemp&i-1);
      call symputx('simactive',simactive);
      call symputx('SimActiveFormat',put(simactive,date7.));
      run;

/* create the dataset to send to SaTScan */
data maven_events;
      set maven.dd_aow_events
            (keep=event_id party_id event_date disease_code disease
            disease_status street_1 x_coord y_coord BORO FIRST_NAME LAST_NAME
            disease_status INVESTIGATION_STATUS event_date GENDER AGE UHF zip
            census_tract_2000);
      event_date = datepart(event_date);
      format event_date mmddyy10.;
      maxtemp = &&maxtemp&i;
      agegroup = "&&agegroup&i";
      x=input(x_coord,8.);
      y=input(y_coord,8.);
      type="Home";
      if disease_code = "&&disease_code&i";
      if "&&agegroup&i" = "AllAges" then output;
      if "&&agegroup&i" = "5orYounger" then do;
            if age <= 5 & age ~= . then output;
      end;
```

```
run;


/* Pull geocoded secondary addresses for events that do not have a */
/*  geocoded address at time of report */
proc sql;
create table secondary_addresses as
      select distinct a.party_id,
                      b.*,
                      case
                            when type_disp="Home (Secondary)" then 1
                            when type_disp="Work" then 2
                            when type_disp="Work (Secondary)" then 3
                      end as hierarchy
      from maven_events a, maven.DD_ADDRESS_HISTORY b
      where a.party_id=b.party_id and a.x_coord is missing and a.y_coord is
missing
            and (index(type_disp,"Work")>0 or type_disp="Home (Secondary)")
            and b.CUSTOM_FIELD1^=" " and b.CUSTOM_FIELD2^=" ";
      quit;

proc sort data=secondary_addresses;by party_id hierarchy descending
end_date;run;

/* Keep most recent geocoded secondary address for each party. */
/* Prioritize, in order: secondary home, primary work, secondary work */
data secondary_address
      (keep=party_id x_coord y_coord street_1 boro zip uhf CENSUS_TRACT_2000
hierarchy);
set secondary_addresses;
      by party_id hierarchy descending end_date;
            if first.party_id;
            rename CUSTOM_FIELD1=x_coord;
            rename CUSTOM_FIELD2=y_coord;
            rename street1=street_1;
            format boro $50.;
            if county="Kings County (Brooklyn)" then boro="BROOKLYN";
            if county="Queens County (Queens)" then boro="QUEENS";
            if county="Richmond County (Staten Island)" then boro="STATEN
ISLAND";
            if county="Bronx County (Bronx)" then boro="BRONX";
            if county="New York County (Manhattan)" then boro="MANHATTAN";
            rename POSTAL_CODE_PREFIX=zip;
            rename CUSTOM_FIELD6=uhf;
            rename tract=CENSUS_TRACT_2000;
      run;

/* Count number of secondary addresses to be added to events dataset */
%let sec_add=%sysfunc(open(secondary_address));
%let num_secondary_address=%sysfunc(attrn(&sec_add,nobs));
%let end=%sysfunc(close(&sec_add));

/* Macro to replace non-geocoded address at time of report */
/*  with geocoded secondary address elements if any exist */
%add_secondary_address(maven_events,secondary_address);
```

```
/*  Pull events from maven_events table: */
/*    1. Exclude non-NYC addresses, contacts/possible exposures, */
/*    Unresolved LEG cases, LEG cases from past outbreaks, and STEC */
/*     cases with Biofire test only */
/*    2.  Recode Boro and Patient Initials */
/*    3.  Format day of week and census tract fields for SaTScan case file */
proc sort data = maven_events; by disease_code maxtemp agegroup; run;
data SatScan1a;
      merge maven_events diseaseListCurrent;
      by disease_code maxtemp agegroup;
      if disease_code = "&&disease_code&i" & disease_status not in
      ('CONTACT', 'POSSIBLE_EXPOSURE') & boro not in ('OUTSIDE NYC' 'NA')
            & event_date >= "&simstart" & event_date <= "&simend";
      if disease_code = "LEG" and disease_status="UNRESOLVED" then delete;
/* Removing LEG cases from period of South Bronx 2015 outbreak */
      if disease_code="LEG" and event_date>='08JUL2015'd and
      event_date<='03AUG2015'd then delete;
/* Removing LEG cases in nursing home nosocomial outbreak */
      if disease_code="LEG" and event_id in("100308152", "100308722",
      "100310829", "100310830", "100310831", "100311061", "100313746") then
      delete;
/* Removing LEG cases from period of outbreak A */
      if disease_code="LEG" and event_date>='25APR2015'd and
      event_date<='15MAY2015'd then delete;
/* Removing LEG cases from period of outbreak B */
      if disease_code="LEG" and event_date>='04NOV2014'd and
      event_date<='31DEC2014'd then delete;
/* Removing LEG cases from period of outbreak C */
      if disease_code="LEG" and event_date>='14SEP2015'd and
      event_date<='21SEP2015'd then delete;
/* Removing STEC cases with positive Biofire test only */
      if disease_code="STEC" and event_id in(&stec_biofire) then delete;
      active = "no ";
      if event_date>="&simActive" then active='yes';
      diseaseName = compress(disease);
      call symput('DiseaseName',diseaseName);
      attrib PtInit length= $5.;
      PtInit = compress(substr(first_name,1,1)||substr(last_name,1,1));
      DOW = weekday(event_date);
/* If census tract is useable, then keep for analysis */
      if census_tract_2000 ^='';
/* Assign state code for use in making 11 digit census tract identifier */
      StateNum=strip('36');
/* Modify Boro for Marble Hill addresses */
      if index(census_tract_2000,'309')~=0 and boro='BRONX' then
Boro='MANHATTAN';
/* Rikers Island men's jails */
      if census_tract_2000 in ('   1','000100') and zip='11370' then
Boro='BRONX';
/* Assign county code for use in making 11 digit census tract identifier */
      if boro = 'BRONX' then BoroCode=strip('005');
      if boro = 'QUEENS' then BoroCode=strip('081');
      if boro = 'BROOKLYN' then BoroCode=strip('047');
      if boro = 'MANHATTAN' then boroCode=strip('061');
      if boro = 'STATEN ISLAND' then borocode=strip('085');
```

```
/* Make 11 digit census tract identifier by compiing state, county, */
/* tract fields */
      CensusTract =
substr(compress(stateNum||BoroCode||census_tract_2000),1,11);
/* Drop individual state, county, tract fields */
      drop census_tract_2000 borocode statenum;
run;

/* Because encephalitis is diagnosed by clinicians (not laboratories), we */
/* restrict encephalitis cases to only those reported by a provider */
/* (eURF=electronic universal reporting form), rather than those whose */
/* only report came from a laboratory. */
      proc sql;
            create table IncludeEnp as
            select distinct disease_code
                  ,event_id
                  ,1 as flag
            from maven.dd_aow_reports
            where reporting_method='EURF' and disease_code='ENP' and
            year(datepart(daterecdbybcd))>=2013
            order by event_id;
      quit;

      proc sql;
            create table IncludeENP2 as
            select s1a.*
                  ,e.flag
            from satscan1a as s1a
            left join includeenp as e
            on e.event_id=s1a.event_id
            order by event_id;
      quit;
      data Satscan1;
            set IncludeENP2;
            if disease_code='ENP' and flag ='' then delete;
      run;

/* Pull in descriptive UHF name to merge with UHF code */
      proc sql;
            create table uhfTable as
            select distinct uhf
                  ,compress(uhfname) as UHFName
            from skt.zip_uhf;
      quit;

/* merge with coordinate file and see if any do not match */
/*  drop them and save the events to an archive file for manual */
/*  address cleaning */
proc import datafile =
      'S:\BCD\COMDISshared\Analyst_of_the_week\Maven\AOW\References\SaTScan\S
      patial variation in data accrual lags\Coordinates
      files/LatLongCoord_for_merging.txt'
      out = coord replace; run;
data coord; set coord; censustract = input(census_tract,$15.); keep
censustract; run;
proc sort data = coord; by censustract; run;
```

```
proc sort data = Satscan1; by censustract; run;
data Satscan1 discordant;
      merge Satscan1 (in=a) coord (in=b);
      by censustract;
      if a & b then output Satscan1;
      if a & ~b then output discordant;
run;
data discordant;
      set discordant;
      keep event_id disease_code boro censustract event_date disease_status;
run;
proc sort data = discordant; by event_id; run;
proc sort data = support.not_merged; by event_id; run;

/* erase the previous dataset on the first loop so fixed records come */
/* off the list */
      %if &i = 1 %then %do;
      data support.not_merged;
            merge discordant (in=a) support.not_merged;
            by event_id;
            if a & disease_code ~= '';
      run;
      %end;
      %if &i ~= 1 %then %do;
      data support.not_merged;
            merge discordant (in=a) support.not_merged;
            by event_id;
            if disease_code ~= '';
      run;
      %end;
      proc sort data=Satscan1;by event_id;run;


/* census tract, date, day of week, # of cases in that census tract */
/* on that day */
      proc sql;
            create table caseFile as
            select censusTract
                  ,event_date
                  ,dow
                  ,count(*) as SatscanCount
            from satscan1
            group by censusTract
                  ,event_date
                  ,dow;
      quit;

/* create case file for SaTScan*/
data _null_;
      set satscan1;
      dummy='1';
      file
      "&TXTFILES.casefile_&&disease_Code&i..&&maxTemp&i.._&&agegroup&i...txt"
      delimiter='09'x DSD DROPOVER lrecl=32767;
      format censusTract $11. ;
      format dummy $1.;
```

```
      format event_date yymmdds10.;
      format dow $1.;
      put censusTract $ @;
      put dummy @;
      put event_date @;
      put DOW;
run;

/* create macro for current disease being run in SaTScan */
      proc sql;
      select distinct quote(trim(disease_code))
            into :disease_code
            from satscan1;
      quit;

/* Define start date, end date, prospective start date, and filenames */
/* for SaTScan run. */
/* "Prospective start date" is an option for adjusting for multiple */
/* testing inherent */
/* in repeated analyses. Even though we do not use this option, the date */
/* must be specified for SaTScan to run */
data _NULL_;
      startdt=put(&simstart, yymmdds10.);
      enddt=put(&simend, yymmdds10.);
      prospstartdate="1900/1/1";*put(&mindate,yymmdds10.);
      file "&PARAM.param.txt";

outfilename="&OUTPUT.SpatialOut_&&disease_code&i..&&maxTemp&i.._&&agegroup&i.
..TXT";
      put

/* Run series of macros read in from SaTScan_macro2 file to */
/*  call SaTScan and produce output */
      %TractParam
      %callSatscan
      %switch
      %if &switch > 0
            %then %do;
                  %CHOROPLETH_setup
                  %linelist_setup
                  %newIndividuals
             %if &NumNew>0 %then %do;
                  %addtolist
                  %MakeChoropleth
                  %MakeClusterLineList
                  %PersonLineList
                  %end;
            %end;
      run;
%end;
%mend RunProgram;

%RunProgram

/* Make archive folders for today and move case files and output files */
/*  from C:\ to daily SaTScan archive folder */
```

```
%macro copyFiles;
%macro dummy; %mend dummy;
options noxwait;
x "md
S:\BCD\COMDISshared\Analyst_of_the_week\Maven\SaTScan\archive\&today\outputTe
xt";
x "move C:\SaTScan9\OUTPUT\*.*
S:\BCD\COMDISshared\Analyst_of_the_week\Maven\SaTScan\archive\&today\outputTe
xt";
x "md
S:\BCD\COMDISshared\Analyst_of_the_week\Maven\SaTScan\archive\&today\casefile
sText";
x "move C:\SaTScan9\TXTFILES\*.*
S:\BCD\COMDISshared\Analyst_of_the_week\Maven\SaTScan\archive\&today\casefile
sText";
x "exit";
%mend copyFiles;
%copyFiles
```

File 3: E-mail Distribution Program

```
/***************************************************************************/
/*    PROGRAM NAME: SaTScanMaster3                                         */
/*    DATE CREATED: February 10, 2014                                      */
/*    LAST UPDATED: November 4, 2015                                       */
/*    PROGRAMMERS: Deborah Kapell                                          */
/*                 Eric Peterson                                           */
/*                                                                         */
/*    PURPOSE: Call SaTScan analysis and associated macros, send           */
/*    reviewer e-mails                                                     */
/*                                                                         */
/***************************************************************************/

OPTIONS NOCENTER  nonumber  ls=140 ps=51 nodate;
options symbolgen;
options mlogic;
options mprint;
options orientation=landscape;

/* If mimicking prospective analysis by running analysis today as if it */
/* were on a prior date (e.g., because IT problems interfered with initial */
/* run), replace today() with the run date you want to simulate. */
/*    Format example: '30JAN2015'd */
data _null_;
call symput ('today',put(today(),date9.));
call symput ('todaynum',today());
call symput ('WeekDay',weekday(today()));
run;

/* Save a permanent text file of the SAS log from each daily run in case */
```

```
/* of problems that require investigation */
proc printto log="S:\BCD\COMDISshared\Analyst_of_the_week\Maven\
                  SaTScan\Archive\logs\Satscan_&today..txt"; run;

%include
'S:\BCD\COMDISshared\Analyst_of_the_week\Maven\SaTScan\SaTScan_v5.sas';run;

/* Events added to SaTScan linelist today */
proc sql; create table NewEvent as
select distinct disease_code
      ,cluster, MaxTemp, agegroup, rundate,
      count(*) as cases
from support.satscanlinelist
where rundate=&todaynum
group by disease_code, maxtemp, agegroup, rundate
order by disease_code, MaxTemp, agegroup, rundate;
quit;

/* Clusters added to the history file today */
proc sql; create table NewCluster as
select distinct disease_code
      ,MaxTemp, agegroup, rundate, NumConfProbSusp, RECURR_INT, recurrence
from support.clusterhistory
where rundate=&todaynum
order by disease_code, MaxTemp, agegroup, rundate;
quit;

/* merge and keep as relevant cluster if one or more new events added to */
/*  the cluster today and meets disease-specific conditions. */
/*  For hepatitis A (HAV), require signals to have >2 conf/prob/susp cases */

data NewEventCluster;
      merge NewEvent (in=a) NewCluster;
      by disease_code MaxTemp agegroup;
      if a & (NumConfProbSusp > 2 | disease_code ~= "HAV");
run;

/* Create macro variables for each row of relevant clusters to loop */
/* through */
data _null_;
      set NewEventCluster;
      by disease_code MaxTemp agegroup;
      if first.disease_code | first.maxTemp | first.agegroup
            then do;
            i+1;
            call symputx('disease_code'||left(put(i,2.)),disease_code);
            call symputx('maxtemp'||left(put(i,2.)),maxtemp);
            call symputx('agegroup'||left(put(i,2.)),agegroup);
            call symputx('end',left(put(i,2.)));
            end;
run;

/* Define as “new cluster” or “new event” signal type */
%macro ClusterEventDefinition;
%macro dummy;
%mend dummy;
```

```
%do i=1 %to &end;

%let
open=%sysfunc(open(existing_&&disease_code&i.._&&maxtemp&i.._&&agegroup&i..))
;
%global num_exist;
%let num_exist=%sysfunc(attrn(&open,nobs));
%let close=%sysfunc(close(&open));

      data NewEventCluster;
      set NewEventCluster;
            format signaltype $10.;
            if disease_code="&&disease_code&i" and maxtemp=&&maxtemp&i and
agegroup="&&agegroup&i" then do;
                  if &num_exist<=0 then signaltype="Cluster";
                  if &num_exist>0 then signaltype="Event";
            end;
      run;

%symdel num_exist;
%end;
%mend ClusterEventDefinition;

%ClusterEventDefinition



/* Counts number of rows in "newclusterevent" dataset, outputs value */
/* to macro variable */
%let dsid=%sysfunc(open(NewEventCluster));
%let numnew=%sysfunc(attrn(&dsid,nobs));
%let rc=%sysfunc(close(&dsid));


/* If no new clusters and no new events, e-mail data staff to */
/*  confirm program ran successfully */
OPTIONS EMAILHOST="app22csmtp" EMAILSYS=SMTP EMAILPORT=25;
%let name = "sgreene4@health.nyc.gov";
%macro noClusters;
%macro dummy;
%mend dummy;
filename mymail
email from="xxxxxxx@health.nyc.gov"
to    =("xxxxxxx@health.nyc.gov")
cc  =("xxxxxxx@health.nyc.gov")
subject="SaTScan: No new clusters today"
emailsys=SMTP;
data _null_;
file mymail;
put 'All quiet.'/
      'FYI, the cluster history is here: \\nasprgshare220\share\DIS\BCD\
            COMDISshared\Analyst_of_the_week\Maven\SaTScan\SupportingFiles'/;
run;
quit;
%mend noClusters;
```

```
/* If new clusters or new events, get reviewer info and e-mail */
/* designated staff */
%macro GetReviewers;
%macro dummy;
%mend dummy;
/* pull reviewer info from existing SAS dataset */
data reviewers2;
      set support.reviewers2;
run;

/* Merge reviewer e-mails with new event and cluster dataset */
proc sql; create table signals_reviewer as
select n.disease_code
      ,n.agegroup
      ,n.maxtemp
      ,n.cluster
      ,n.signaltype
      ,n.cases
      ,r.notes as reviewer
from NewEventCluster as n
      ,reviewers2 as r
where n.disease_code=r.code
order by reviewer;
quit;

proc sort data=signals_reviewer nodupkey;by reviewer maxtemp disease_code
signaltype;run;

/* Split into separate datasets by new clusters and events */
data signals_reviewer_clusters signals_reviewer_events;
set signals_reviewer;
      if signaltype="Cluster" then output signals_reviewer_clusters;
      if signaltype="Event" then output signals_reviewer_events;
run;

/* Split events of some diseases into separate datasets for multiple */
/* analysis parameters: */
/* AllAges and 5andunder - amebiasis (AMB), */
/* cryptosporidiosis (CSP), giardiasis (GIA) */
data signals_reviewer_REST signals_reviewer_AMBCSPGIA;
set signals_reviewer_events;
      if disease_code ^in("AMB","CSP","GIA",)
            then output signals_reviewer_REST;
      if disease_code in("AMB","CSP","GIA")
            then output signals_reviewer_AMBCSPGIA;
run;

/* Keep one row per unique disease-reviewer combination */
proc sort data=signals_reviewer_clusters nodupkey;by disease_code
reviewer;run;

/* Counts number of rows in "newcluster" dataset, output to macro variable */
%let dsid2=%sysfunc(open(signals_reviewer_clusters));
%global numnew_unique_clusters;
%let numnew_unique_clusters=%sysfunc(attrn(&dsid2,nobs));
```

```
%let rc2=%sysfunc(close(&dsid2));

/* Counts number of rows in "newevents_REST" dataset, output to */
/* macro variable */
%let dsid3=%sysfunc(open(signals_reviewer_REST));
%global numnew_unique_REST;
%let numnew_unique_REST=%sysfunc(attrn(&dsid3,nobs));
%let rc3=%sysfunc(close(&dsid3));

/* Counts number of rows in "newevents_AMBCSPGIA" dataset, output to */
/* macro variable */
%let dsid4=%sysfunc(open(signals_reviewer_AMBCSPGIA));
%global numnew_unique_AMBCSPGIA;
%let numnew_unique_AMBCSPGIA=%sysfunc(attrn(&dsid4,nobs));
%let rc4=%sysfunc(close(&dsid4));



proc sort data=signals_reviewer_REST;by disease_code maxtemp reviewer;run;
proc sort data=signals_reviewer_AMBCSPGIA;by disease_code agegroup
reviewer;run;

%mend GetReviewers;

/* If there are *new clusters,* this macro will e-mail designated BCD */
/* staff and cc data staff and BCD leadership */
%macro ClustersExist;
%macro dummy;
%mend dummy;

%if &numnew_unique_clusters=0 %then %return;

proc sort data=signals_reviewer_clusters;by disease_code reviewer;run;

data _null_;
      set signals_reviewer_clusters;
      by disease_code;
      if first.disease_code
            then do;
            i+1;
            call symputx('disease'||left(put(i,2.)),disease_code);
            call symputx('reviewer'||left(put(i,2.)),reviewer);
            call symputx('end',left(put(i,2.)));
            end;
run;

%do i=1 %to &numnew_unique_clusters;
data reviewer&i;
      set signals_reviewer_clusters;
      where disease_code="&&disease&i" and reviewer = "&&reviewer&i";
run;

filename mymail
email from="xxxxxxx@health.nyc.gov"
to    =(&&reviewer&i)
cc  = ("xxxxxxx@health.nyc.gov")
```

```
subject="SaTScan: New &&disease&i signal"
emailsys=SMTP;

data _null_;
file mymail;
put "New SaTScan signal for &&disease&i on &today"//
      "SaTScan cluster information is here:"/
      "\\nasprgshare220\share\DIS\BCD\COMDISshared\Analyst_of_the_week\
            Maven\SatScan\archive\&today\"//
      "If you have any questions, please ask an analyst."/;
run;
quit;
%end;
%mend ClustersExist;



/* If there are *new events* added to ongoing clusters, this macro will */
/* e-mail designated BCD staff */
%macro EventsExist;
%macro dummy;
%mend dummy;

%if &numnew_unique_REST=0 %then %return;

data _null_;
      set signals_reviewer_REST;
      by disease_code maxtemp reviewer;
      if first.disease_code
            then do;
            i+1;
            call symputx('disease'||left(put(i,2.)),disease_code);
            call symputx('maxtemp'||left(put(i,2.)),maxtemp);
            call symputx('reviewer'||left(put(i,2.)),reviewer);
            call symputx('newcases'||left(put(i,2.)),cases);
            call symputx('end',left(put(i,2.)));
            end;
run;

%do i=1 %to &numnew_unique_REST;
data reviewer&i;
      set signals_reviewer_REST;
      where disease_code="&&disease&i" and maxtemp=&&maxtemp&i and
reviewer="&&reviewer&i";
run;

filename mymail
email from="xxxxxxx@health.nyc.gov"
to    =(&&reviewer&i)
cc  = ("xxxxxxx@health.nyc.gov")

subject="SaTScan: Ongoing &&disease&i signal"
emailsys=SMTP;

data _null_;
```

```
file mymail;
put "&&newcases&i cases of &&disease&i added to ongoing SaTScan signal on
&today."//
      "SaTScan cluster information is here:"/
      "\\nasprgshare220\share\DIS\BCD\COMDISshared\Analyst_of_the_week\
            Maven\SatScan\archive\&today\"//
      "If you have any questions, please ask an analyst."/;
run;
quit;
%end;
%mend EventsExist;


/* If there are new AMB,CSP,GIA events this macro will e-mail */
/* designated BCD staff */
%macro AMBCSPGIAEventsExist;
%macro dummy;
%mend dummy;

%if &numnew_unique_AMBCSPGIA=0 %then %return;

data _null_;
      set signals_reviewer_AMBCSPGIA;
      by disease_code agegroup reviewer;
      if first.agegroup
            then do;
            i+1;
            call symputx('disease'||left(put(i,2.)),disease_code);
            call symputx('agegroup'||left(put(i,2.)),agegroup);
            call symputx('reviewer'||left(put(i,2.)),reviewer);
            call symputx('newcases'||left(put(i,2.)),cases);
            call symputx('end',left(put(i,2.)));
            end;
run;

%do i=1 %to &numnew_unique_AMBCSPGIA;
data reviewer&i;
      set signals_reviewer_AMBCSPGIA;
      where disease_code="&&disease&i" and agegroup="&&agegroup&i" and
reviewer="&&reviewer&i";
run;

filename mymail
email from="xxxxxxx@health.nyc.gov"
to    =(&&reviewer&i)
cc   = ("xxxxxxx@health.nyc.gov")

subject="SaTScan: Ongoing &&disease&i signal"
emailsys=SMTP;

data _null_;
file mymail;
put "&&newcases&i cases of &&disease&i (&&agegroup&i) added to ongoing
SaTScan signal on &today."//
      "SaTScan cluster information is here:"/
      "\\nasprgshare220\share\DIS\BCD\COMDISshared\Analyst_of_the_week\
```

```
            Maven\SatScan\archive\&today\"//
      "If you have any questions, please ask an analyst."/;
run;
quit;
%end;
%mend AMBCSPGIAEventsExist;




/* All e-mail macros run together */
%macro decision;
%if &numnew=0
      %then %do;
            %noclusters
      %end;
%if &numnew>0
      %then %do;
            %GetReviewers
            %if &numnew_unique_clusters>0 %then %do;
                  %ClustersExist
            %end;
            %if &numnew_unique_REST>0 %then %do;
                  %EventsExist;
            %end;
            %if &numnew_unique_AMBCSPGIA>0 %then %do;
                  %AMBCSPGIAEventsExist;
            %end;
      %end;
%mend decision;

%decision

proc printto; run;
```
